# Rapid and Resilient LoRa Leap: A Novel Multi-Hop Architecture for Decentralised Earthquake Early Warning Systems

**DOI:** 10.3390/s24185960

**Published:** 2024-09-13

**Authors:** Vinuja Ranasinghe, Nuwan Udara, Movindi Mathotaarachchi, Tharindu Thenuwara, Dileeka Dias, Raj Prasanna, Sampath Edirisinghe, Samiru Gayan, Caroline Holden, Amal Punchihewa, Max Stephens, Paul Drummond

**Affiliations:** 1 Department of Electronic & Telecommunication Engineering, University of Moratuwa, Moratuwa 10400, Sri Lanka; ranasingheradvc.19@uom.lk (V.R.); udaraagn.19@uom.lk (N.U.); mathotaarachchimm.19@uom.lk (M.M.); thenuwarata.19@uom.lk (T.T.); dileeka@uom.lk (D.D.); samirug@uom.lk (S.G.); 2 Joint Centre for Disaster Research, Massey University, Wellington 6021, New Zealand; 3 Department of Computer Engineering, University of Sri Jayewardenepura, Ratmalana 10390, Sri Lanka; essedirisinghe@sjp.ac.lk; 4 SeismoCity Ltd., Wellington 6023, New Zealand; caroline.holden@seismocity.co.nz; 5 ADP Consultancy, Palmerston North 4410, New Zealand; amal_yahu@yahoo.com.sg; 6 Civil and Environmental Engineering, Faculty of Engineering, University of Auckland, Auckland 1023, New Zealand; max.stephens@auckland.ac.nz; 7 Canterbury Seismic, Christchurch 8442, New Zealand; paul.drummond@canterburyseismic.com

**Keywords:** earthquake, multi-hop, broadcast, delay, PLUM, EEW, LoRa, FLoRa

## Abstract

We introduce a novel LoRa-based multi-hop communication architecture as an alternative to the public internet for earthquake early warning (EEW). We examine its effectiveness in generating a meaningful warning window for the New Zealand-based decentralised EEW sensor network implemented by the CRISiSLab operating with the adapted Propagation of Local Undamped Motion (PLUM)-based earthquake detection and node-level data processing. LoRa, popular for low-power, long-range applications, has the disadvantage of long transmission time for time-critical tasks like EEW. Our network overcomes this limitation by broadcasting EEWs via multiple short hops with a low spreading factor (SF). The network includes end nodes that generate warnings and relay nodes that broadcast them. Benchmarking with simulations against CRISiSLab’s EEW system performance with internet connectivity shows that an SF of 8 can disseminate warnings across all the sensors in a 30 km urban area within 2.4 s. This approach is also resilient, with the availability of multiple routes for a message to travel. Our LoRa-based system achieves a 1–6 s warning window, slightly behind the 1.5–6.75 s of the internet-based performance of CRISiSLab’s system. Nevertheless, our novel network is effective for timely mental preparation, simple protective actions, and automation. Experiments with Lilygo LoRa32 prototype devices are presented as a practical demonstration.

## 1. Introduction

### 1.1. Background

Across the world, with rapidly increasing urbanisation, earthquakes (EQs) pose a serious threat to lives and properties in areas near major active faults [1]. Sensor and telecommunication technologies supporting (1) post-earthquake information on buildings and other critical infrastructure and also (2) real-time earthquake early warning (EEW) and near-real-time EQ information have been identified as crucial for enhancing the resilience of the members of our society and infrastructure [2]. 

Among the above-described technology solutions, EEW systems (EEWSs) can contribute towards the psychological preparedness of the members of our communities to anticipate earthquake shaking and also support them in performing simple drop-cover-hold safety actions [3]. In addition, EEW can also support automation and preprogramming of systems to take emergency measures [3]. However, two key factors, (1) extreme complexity involved in the occurrences of earthquakes and (2) limited availability or failures of communication infrastructures, make it challenging for such technologies to become reliable and sustainable [4]. Despite these challenges, there have been several advancements in accurate measures of ground shaking with the advances in seismic instrumentation, digital communication, algorithms and processing [5].

Across the globe, several countries and territories, such as Japan, the USA, Taiwan, and Mexico, have successfully implemented national-level officially authorised EEW services [6,7,8,9,10]. These systems demonstrate the ability to provide valuable seconds of warning about the ground shaking due to an EQ, leading to support members of the community by preparing them physically and mentally for anticipated ground shaking and helping reduce impacts [11,12].

Despite its advantages, the implementation of EEWSs on a national scale has encountered significant technical and non-technical challenges. Among these challenges, extreme implementation costs [4,13,14,15] and high operating and maintenance costs are the most critical [11]. Therefore, spending on high-end EEW at a national scale may not be economically viable even in countries considered prone to large-scale EQs [4,16]. These challenges can lead to the viability of such systems, particularly for developing countries, and can be unviable even for a developed country like Aotearoa, New Zealand (NZ).

There have been a number of promising attempts at low-cost solutions supported by novel technological approaches to address the challenges of the described extreme costs associated with typical national-level EEW solutions worldwide, particularly the solutions driven by micro-electromechanical system (MEMS)-based ground motion sensors [9]. Although introduced as a potential technology in the 1990s [17], accurate detection of EQs using MEMS-based accelerometers showed promising results more recently [4]. Among such solutions, MEMS-based EEW systems in Taiwan [18], China [19], and India [20] have demonstrated their applicability for real-life use to alert the members of the public. In addition to such working systems, there have been several notable MEMS-based experimental systems or working prototypes operating in countries like the US [21], Iceland [22], and NZ [4,5]. The decreasing cost of producing hardware and their increased performance have made these MEMS-based systems evolve into more robust yet affordable EEW solutions, demonstrating their ability to either work in a complementary fashion with traditional high-cost EEWSs or operate as standalone EEWSs [4]. With appropriate R&D and government support, these systems have great potential to provide EEW solutions for countries vulnerable to earthquakes that may not be financially capable of affording high-end EEWSs [5]. 

The end-to-end data communication needs of an EEW system can be broadly divided into two parts: data communication needs required for (1) EQ detection and warning generation and (2) warning dissemination. Independent of the type or the quality of the sensors, EEW systems researched and implemented across the world tend to transmit either raw or minimally processed data captured from their seismometers to a remotely located central server, usually on a cloud-based server [23], which runs algorithms to detect EQs in a more centralised manner [4,5]. Processing EQ data in such a manner at centrally located servers creates several technological vulnerabilities and limitations despite the advantage of having better control over data processing and consistent detection, collection, and processing of data during a disaster and immediately afterwards [5]. On several occasions, the EEW literature has identified the vulnerabilities that could be generated due to high dependency on centralised processing and highlighted the importance of having a strategy for redundancy technologies as backup solutions for data communication and processing [14,18,24,25]. Such solutions may include multiple central servers located at different locations and satellite-based data communication [25]. Despite investment in redundancy solutions, EEW systems remain vulnerable to providing a reliable service more sustainably due to the potential loss of internet access as a result of failures of telecommunication networks after a large earthquake [5]. Such vulnerabilities can disrupt the performance of EEW networks, undermining the effectiveness of warnings and highlighting the need for further innovation in more resilient communication solutions [24].

### 1.2. MEMS-Based Decentralised EEW Network

Having recognised the above technology gap, a few years back in 2022, we took the first step towards minimising the dependency of EEW systems on centralised processing by introducing a novel approach to generating EEWs by using low-cost Raspberry Shake 4D sensors (RS-4D), a MEMS-based low-cost ground motion sensor manufactured by Raspberry Shake, S.A. in Panama [4]. With our approach, RS-4D sensors were installed in the homes of members of our communities in NZ. The sensors were connected in a virtual mesh network driven by SD-WAN-based hole-punching architecture to detect EQs and generate EEWs [4]. In this network, the captured ground motion data were processed in a decentralised manner at the sensor nodes by running EQ detection algorithms. Having detected EQs, EEW generation could occur at a sensor node and act as the point of warning dissemination [4]. This EEWS employed a well-known ground motion-based EQ detection algorithm, which is simple yet robust [26].

To our knowledge, only minimal research is reported in the literature that promotes a decentralised approach to EEW. This includes the decentralised EEW architecture proposed by [27], which reported a simulation of machine learning-based EQ detection at the sensor nodes. The simulated results reported demonstrate the feasibility of the decentralised approach to EEW. However, this EEW network implementation is more conceptual in nature implemented as a simulation in a single computer and not a real-world deployment. In comparison, the fully decentralised EEW architecture proposed by us [4] can be considered an end-to-end EEWS implementation with actual RS-4D sensors deployed at people’s homes that fully detect EQs and generate and disseminate EEWs at the RS sensor nodes [5]. Unlike other traditional EEWSs, ours runs a robust ground motion-based EQ detection algorithm at the node level rather than centrally [5]. Having been implemented as an experimental EEWS, this system has demonstrated evidence of the performance of this novel approach using EQ data captured from the RS-4D ground motion sensors [4,5]. This work claims that an RS-4D sensor network driven by node-level data processing can outperform traditional centralised processing in terms of system latency, redundancy, and implementation cost [5].

### 1.3. PLUM-Based EQ Detection and Alert Generation in the MEMS-Based EEW Sensor Network

As described above, the low-cost MEMS-based EEW network implemented by the authors utilises an adapted version of PLUM (Propagation of Local Undamped Motion), a ground motion-based EQ detection algorithm that has come to the fore recently [26]. PLUM is considered a robust yet lightweight threshold-based algorithm capable of predicting the seismic intensities at the given prediction points within an area of a 30 km radius [26]. Thus, we found the PLUM algorithm to be the most appropriate EQ detection algorithm to operate successfully within our decentralised, MEMS-based EEW architecture. With the original PLUM approach, the observation sensors continuously predict the shaking intensities for a prediction sensor located at a known, predefined location, around which a 30 km circle is defined for observation sensors. In our approach, while maintaining a threshold-based trigger of PLUM, we define a 30 km circle around the sensor that first detects the EQ by running a simple threshold-based ground motion EQ detection algorithm. Thus, in our adapted PLUM approach, circle locations are dynamic and depend on where the EQ is first detected. Immediately after detecting an EQ for the first time, it will share an unverified alert among the other sensors. 

The PLUM algorithm operates with a single operating point. To improve the reliability of EQ detection and to minimise the anticipated false alarms by having only a single observation station, we introduced a two-station trigger concept to the PLUM algorithm [28]. This two-station trigger concept can reduce the number of false alerts with a PLUM-driven EQ detection and increase the accuracy of the alert generation [5]. With the two-station trigger combined with the adapted PLUM approach, a sensor within a dynamically defined 30 km circle that has already received an unverified alert will move to a listening mode. Subsequently, if that sensor received an actual EQ and managed to detect it after having successfully triggered the threshold-based algorithm within a certain predefined time window from the receipt of that unverified alert, then a verified alert or, in other words, actual EEW will be issued to the rest of the sensors within that 30 km radius circle. This verified warning will be received and accepted as an EEW if any of those remaining sensors do not detect an EQ within a given period of time. Otherwise, the system will terminate the alert. Please refer to Section 8.2 for details on end-to-end EQ detection, alert generation, and dissemination. Importantly, all alerts are embedded with a unique identifier (ID) in their payload that can identify which sensor triggered the alert. In addition, each sensor stores a table of sensor IDs and their physical locations. With that, at any given point in time, each sensor will know the distance between the sensors that generate the alert and support maintaining the shaking intensity boundary required for the PLUM approach. 

### 1.4. LoRa as an Alternative Communication Platform for the Decentralised EEW Network

While having highly decentralised node-level processing of data avoids a single point of failure, which is highly likely when having centralised processing of ground motion data at remote servers [5] dra, our decentralised sensor network described above requires access to the internet to transmit data between sensors and hence is still vulnerable to failure of telecommunication infrastructure. Therefore, this decentralised EEW architecture may not be able to detect earthquakes consistently or generate early warnings during large-scale events. Without internet coverage, this network cannot provide warnings to isolated rural areas, especially for aftershocks when telecommunications are disrupted at the beginning of a severe earthquake sequence.

To address this crucial limitation, we propose a novel LoRa-based multi-hop broadcast network for decentralised EEWSs whose sensor nodes are placed in participating community households. Our goal is to transmit alert messages using LoRa to all nodes within a 30 km radius before the S-wave reaches them. As presented in Section 2.2 and Section 2.3, we conducted a literature review to explore the state-of-the-art use of LoRa in multi-hop/mesh configurations and LoRa for emergency communications. Supported by the literature review findings, this is the first study with such an architecture for LoRa and for disseminating messages across a large area with strict latency constraints.

LoRaWAN [29] is a popular open standard for Low-Power Wide-Area Network (LPWAN) technologies. It is a widely used wireless technology in applications requiring low-power operation and long range at the cost of latency. It is typically used in a star topology, facilitating communication between end devices and a gateway which connects to a central application server. While the open standard defines the medium access control (MAC) layer and the network topology, the physical layer is based on LoRa (long range), a proprietary modulation technique which uses Chirp Spread Spectrum (CSS) for radio modulation [29]. The data signal is modulated onto a chirp signal that increases or decreases its frequency with time. The chirp rate in chirps/s equals the spectral bandwidth (BW) of a LoRa signal, i.e., one chirp per second per Hz of bandwidth. Channel bandwidths used typically are 125, 250, and 500 kHz. The spreading factor (SF), which varies between 7 and 12, is the number of raw bits carried per symbol. A LoRa symbol is composed of 2*^SF^* chirps. A forward error correction (FEC) scheme is used with rates of 4/5, 4/6, 4/7, and 4/8. Interesting trade-offs between SF, the communication range, and the transmission time (or conversely, the data rate) are available when choosing the above key parameters for applications. 

The availability of devices at affordable cost and proven performance in LPWAN IoT applications make LoRa an attractive choice for a communication platform independent of public infrastructure. While satellite technologies are excellent alternatives that are resilient to terrestrial disaster situations, they are still too expensive to reach individuals. 

The remainder of this paper describes our exploration of answering the research question, “Can a LoRa-based multi-hop data communication approach support reaching latency levels required for an EEWS that operates with fully decentralised EQ detection, warning generation, and dissemination and supports uninterrupted service?”. 

The main contributions of this paper are as follows:

1. Introduction of a novel multi-hop broadcast network architecture based on the LoRa physical layer.
2. Development of network node hardware and demonstration of the proposed architecture in a scaled-down field experiment as proof of concept.
3. Extension of a LoRaWAN simulation platform to enable multi-hop broadcast network simulation.
4. Analysis of the performance of the proposed network to evaluate its capabilities for message dissemination.
5. Evaluation of the proposed network as applied to a real-life EEW system established in the Greater Wellington region in NZ.

Section 2 of this paper presents related work on LoRa/LoRaWAN-based multi-hop or mesh configurations and emergency communications. Section 3 provides an overview of LoRa and its performance in relation to its key parameters and trade-offs relevant to our study. Section 4 and Section 5 introduce the proposed multi-hop network architecture and the simulation platform, respectively. Section 6 details the network design steps, followed by Section 7, which evaluates network performance. Section 8 compares the LoRa-based multi-hop architecture’s performance with a previous NZ-based case study involving six EQ scenarios in Wellington. This paper concludes by discussing findings, contributions, limitations, and future work.

## 2. Related Work

After a brief review of LoRa/LoRaWAN applications, we present below a review of recent work on LoRa-based mesh and multi-hop networks and on LoRa/LoRaWAN for disaster-related communications.

### 2.1. LoRa/LoRaWAN Applications

Of the four dominant Low-Power Wide-Area Network (LPWAN) technologies in current use, LoRaWAN, Sigfox, NB-IoT, and LTE-M, LoRaWAN is one of the most promising solutions where long-range and low-power operations are essential and where communication infrastructure is absent. LoRa transceivers embedded in LoRaWANs may run for up to ten years on battery power [30].

LoRaWANs are typically used for applications such as asset monitoring and management, infrastructure monitoring, smart cities, and industrial IoT deployments [31] provide a comprehensive review of smart city applications enabled by LoRa in a range of domains, including agriculture, energy, environment, healthcare, industry, transportation, and waste management. Monitoring smart water grids (SWGs) and sanitation systems in [32,33], respectively, sensors for measuring parameters in agriculture [34], and forest fire detection [35] are some examples of reported LoRaWAN applications that exemplify the long-range, low-power requirements and the infeasibility of using cellular systems or the internet. This contrasts with wireless wide-area networks typically used by the general public or large corporate organisations with high-speed data and no power constraints.

### 2.2. Multi-Hop/Mesh LoRa/LoRaWAN Systems

There have been multiple attempts to build multi-hop/mesh networks with LoRaWAN. One of the widely known projects is Meshtastic^®^ [36], where each radio in the network is designed to rebroadcast the messages they receive. The current implementation of the project allows 100 concurrent devices and has demonstrated around a 254 km range. Meshtastic^®^ is a community-driven project which supports a wide range of off-the-shelf hardware platforms such as RAK Meshtastic Start Kit, Station G1, LILYGO LoRa T3-S3, and HELTEC LoRa V3. Besides projects like Meshtastic^®^, several other research studies have presented different ways to build multi-hop/mesh networks using LoRaWAN [37]. 

Farooq [38] investigated the use of multi-hop communication along with the fastest data rate setting to exactly match the coverage of the setting recommended by LoRaWAN. This work demonstrates that a multi-hop configuration with two intermediate relay nodes (three hops) at the fastest data rate setting provides better reliability, lower energy consumption, and significantly lower end-to-end latency for the same range as the LoRaWAN recommended settings. 

Lee and Ke [39] observed that LoRa networks struggle to reliably deliver packets in urban areas. Instead of adding more gateways, the authors studied a mesh network architecture to increase the packet delivery ratio. This study demonstrated the superiority of mesh networks in performance. Primarily, when the network supports more than three hops, the packet delivery ratio demonstrates a significant improvement. 

The work presented by Huh and Kim [40] shows a private LoRa mesh network that supports time-division multiple access. The proposed architecture allows the user nodes to cooperate with each other to deliver a packet to a gateway. The cooperation between nodes facilitates self-configuration of the network for optimum packet routing. Moreover, the authors introduced a time-slotted event-driven system to battle the packet collision issues if there are a large number of nodes in the first hop. With these mechanisms, they successfully demonstrated the application of the proposed network in fire emergencies, streetlamps, and toxic gas detection systems.

The study reported by Almeida et al. [41] demonstrates a hybrid LoRa mesh/LoRaWAN network. The authors designed a novel LoRa mesh network and a routing algorithm to cater to geographical areas that cannot be covered with regular LoRaWAN networks. The LoRa mesh network is coordinated by a proxy node which falls in the LoRaWAN coverage area. The proxy node uses a simplified version of the ad hoc on-demand distance vector (AODV) algorithm for routing, and the experimental results show a 15 s duration for the route creation in the LoRa mesh network.

Berto et al. [42] present a LoRa-based mesh network targeting peer-to-peer communication with no gateways. The study presents a well-designed protocol stack consisting of three layers: (i) physical; (ii) link, network, transport; and (iii) application. The system was implemented in an ESP32 and an SX1276 transceiver. End nodes use an internal matrix-based routing table for forwarding messages and run web servers that can respond to requests from other nodes. Requests are addressed to specific destination nodes. The experiments that observed the single-hop vs. double-hop latency demonstrated significant differences, such as 524 ± 93 ms vs. 863 ± 109 ms for 250 kHz bandwidth, respectively. Similarly, with reduced bandwidth (125 kHz), the latencies are 9315 ± 56 ms and 18,636 ± 308 ms, respectively. However, the experiments conducted to observe the range indicate that the reduced bandwidth options perform, well while the broadband option fails at very short distances, such as 1.2 km.

Mai and Kim [43] proposed a multi-hop LoRa network protocol that is collision-free with low latency. A tree topology is constructed by exchanging packets between LoRa and sink nodes. During this period, a timeslot and channel are assigned to each tree link, over which LoRa nodes communicate with their parent node and which are collision-free with their neighbour nodes.

While LoRaWAN has been successfully deployed in numerous IoT applications, there are many applications that would benefit from more flexible network topologies than its star of stars, according to Centelles et al. [44]. This research investigates the effects of adding multi-hop capability to LoRaWAN as a strategy to overcome gateway infrastructure failures, such as coordinated response in the aftermath of natural disasters such as an earthquake. Nodes can communicate with each other without a gateway. A minimalistic distance-vector routing protocol is designed to forward packets to a specific destination with a reduced end-to-end transmission time. This research is extended to the architecture named LoRaMoto [45] that can be used in post-disaster scenarios to establish civilian communication. The LoRaMoto system would enable people to communicate short messages with their families and emergency response teams. The simulation studies conducted with the proposed architecture showed that the system can be scaled up to a few thousand nodes. However, the system starts to fail beyond more than ten thousand nodes. 

### 2.3. LoRa for Emergency Communications

Although not directly focused on providing solutions for EEW, a considerable amount of research has been conducted in the last decade exploring LoRa for communication during emergencies. 

Esposito et al. [46] comprehensively reviewed IoT solutions in early warning (EW) systems for various natural disasters. They explored LoRa as a potential communication method alongside other LPWAN technologies like Sigfox and NB-IoT, 3G/4G/5G cellular networks, and short-range technologies such as WiFi, BLE, and IEEE802.15.4. While LoRa’s advantages are identified, they note the limitation of using a shared unlicensed spectrum.

Wang et al. [47] proposed a novel real-time landslide monitoring method based on LoRa and an intelligent adaptive sensing Internet of Things (IoT) concept. In the normal mode of operation, a low-speed clock is used for data collection as an energy-saving measure. When the data meet a trigger condition, the system enters a high-speed data collection mode. A LoRaWAN gateway relays information from the data collection nodes via a cellular network to a cloud server and a central management platform.

In research conducted by Manuel et al. [48], a LoRaWAN architecture for search-and-rescue missions was developed and tested. They introduced a Search-and-Rescue Robot (SAR) equipped with a novel full-duplex LoRa-based communication device that receives control commands from and sends its location to a base station. They managed to achieve a range of 1.6 miles but did not investigate system latencies. 

Macaraeg et al. [49] proposed a LoRa-based mesh network for emergency communication. To enable mesh functionality, a modified ad hoc on-demand distance vector (AODV) routing protocol with the received signal strength indicator (RSSI) as the routing metric was presented. One-hop and two-hop packet delivery rates (PDR) were tested. PDR performance deteriorates with increasing hop count, especially at higher SFs. Latencies were not studied.

Sisinni et al. [50] proposed a LoRa-REP replication scheme to handle critical messages in industrial plants after emergencies, increasing reliability and reducing latency. A critical event triggers the transmission of multiple replicas of a message on the uplink, each using a different SF, all within the transmit window. Acknowledgements are received during corresponding receive slots. To further reduce latency, an enhanced LoRa-REP physical layer using SDR devices is designed for simultaneous transmission of frames with different SF values. Each message appears to the LoRaWAN back-end as coming from a different virtual node.

Dalpathadu et al. [51] presented a solution suitable for post-disaster rescue communications based on LoRa. They managed to disseminate data between rescuers using the concepts of opportunistic networks and employing the epidemic forwarding protocol. Message delivery delays in the range of 25–100 s were reported.

Centelles et al. [52] proposed a system that uses the LoRaWAN architecture to allow citizens to report their status to emergency units and public authorities with simple messages and interaction mechanisms following an earthquake. LoRa radio technology is used to transmit information between the users’ nodes in their homes and workplaces, and an application is hosted in the LoRa network server via gateways. These nodes are also able to receive downlink messages and display notifications. A realistic environment is modelled with the FLoRa simulator [53], and user interaction for a 120 s period is studied following an earthquake event. A best-case packet delivery rate of 50–50% was reported, and it was seen that communication in the system does not scale well.

Tsamakis et al. [54] applied machine learning methods to facilitate the transmission of event packets containing critical information with low delay to a central server through a LoRaWAN architecture. A MAC protocol selection scheme that depends on the network traffic load was presented instead of the LoRaWAN MAC. An example application considered was the detection of forest fires with a maximum response time limit set at approximately 10 min.

Navarro-Ortiz et al. [55] proposed a self-healing LoRaWAN network architecture in order to provide resilience in disaster situations. It addresses the possible faults of core network elements, and resilience is achieved by microservice orchestration with several replicas of the LoRaWAN network entities and a load balancer.

### 2.4. Key Take-Aways from Related Work

Of the studies on multi-hop approaches, most adopt packet delivery rate (PDR) as the performance measure, while [38,42,52] investigate latency aspects as well. They demonstrate low-latency, long-range communications via multiple hops in LoRaWAN. These encourage us to examine the potential for the extension of LoRa to a multi-hop broadcast network architecture based on its physical layer for applications with concurrent long-range and critical latency requirements, such as EEW dissemination. The concept is further motivated by our requirement to reach every peer node in the network. This is in contrast to reaching a gateway as in [38], reaching a specific destination as in [42], between a source–destination pair as in [52], or between previously identified nodes as in [51]. Broadcasting will contribute to faster message traversal through the network and provide multiple routes that enhance resilience, simplicity, and scalability. However, flooding the network in this manner may cause packet losses due to collisions, as highlighted in [56], where link saturation and buffer overflow in nodes need careful examination. 

Despite a number of disaster management-related applications that use LoRa, there is very limited or no exploration of its investigation as an alternative solution for early warnings with a time-critical requirement such as EQs. Although there have been successful LoRa implementations for post-emergency scenarios to transmit data between devices in mesh network environments [36,51], the speeds achieved in such approaches cannot meet the latency requirements expected for EEW solutions. These efforts focus on LoRa as an access technology when conventional infrastructure is unavailable during post-disaster situations. Additionally, many use LoRaWAN to convey disaster information to a central entity. Further, none of the studies reported sub-10 s latencies as a performance requirement. 

### 2.5. Motivation for the Research

The motivation for research into low-latency communication with LoRa stems from the need to develop solutions that meet both long-range and critical latency requirements for applications such as earthquake early warning (EEW) dissemination. Of the previous studies on multi-hop LoRa communications, those that examined low-latency communications have demonstrated the potential. However, their implementations do not consider a network architecture that could serve multiple peer nodes simultaneously and where it is critical to reach multiple nodes in a time-sensitive manner.

This gap presents an opportunity to explore how and to what extent LoRa can be extended to a multi-hop broadcast architecture, which could potentially offer faster message traversal through the network, increased resilience, and enhanced scalability. 

However, our work does not aim to compete with the high speeds or low latencies achievable by technologies like 5G, especially in ultra-reliable low-latency communication (URLLC) scenarios such as vehicular communication, virtual reality, or gaming. Rather, we focus on exploring how LoRa’s long-range capabilities can be effectively utilised to deliver timely communication without relying on wide-area public telecommunications infrastructure.

## 3. LoRa Characteristics and Trade-off Analysis

The LoRa PHY layer, as briefly introduced in Section 1.4, supports several settings that impact communication range (coverage), reliability (resilience), and energy consumption. Table 1 summarises the two extreme SF settings of LoRa. It was shown by [57] that the achievable range with setting A of PHY parameters recommended for LPWAN applications is nearly three times that achievable with setting B, which gives the fastest data rate. Farooq [38] demonstrated that three hops with setting B provide significantly lower end-to-end latency for the same range as the LoRaWAN recommended setting A. In between these extreme settings, there is a multitude of options with varying degrees of range, latencies, and data rates. This section provides an overview of LoRa’s physical layer characteristics and an analysis of the possible trade-offs relevant to our application across the range of available SFs.

### 3.1. Time on Air (ToA)

**Figure 1:**
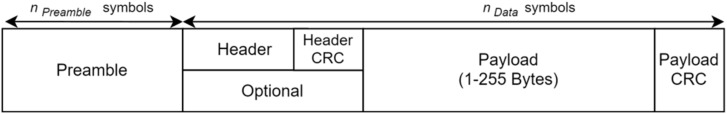
LoRa frame format (Source: [58]).

A LoRa symbol is composed of 2*^SF^* chirps, which cover the entire frequency band. A LoRa frame consists of multiple symbols as shown in Figure 1. The ToA is the time taken for the transmission of one frame. 

The symbol duration is given by [58]

(1) $$T_{S}=\frac{2^{SF}}{BW}$$

Assuming a fixed *BW*, the data rate can change depending on the employed spreading factor (*SF*). Thus, the data rate *R_b_* is calculated as

(2) $$R_{b}=SF\times \left(\frac{BW}{2^{SF}}\right)\times \left(\frac{4}{4+CR}\right)$$

where the second term corresponds to *R_S_*—the symbol rate (symbols/s)—and the third term depends on the forward error correction (FEC) scheme used. Thus, assuming a fixed *BW* and coding rate, the data rate decreases as the *SF* increases. 

As illustrated in Figure 1, the LoRa frame consists of a preamble, an optional header, and the data payload. The LoRa preamble is, by default, an eight-symbol-long sequence. This is followed by an optional header. The header, when present, is transmitted with a maximum error correction code (4/8). It also has its own CRC to allow the receiver to discard invalid headers. The header indicates the size of the payload (in bytes), the code rate used for the payload, and the presence of an optional 16-bit CRC for the payload. If present, this CRC is appended after the payload. In certain scenarios, where the payload, coding rate, and CRC presence are fixed or known in advance, it may be advantageous to reduce transmission time by invoking the implicit header mode. In this mode, the frame has no header. 

The time on air (ToA) of a LoRa frame is a crucial performance measure in a latency-critical application such as the one considered in this paper. 

Considering the LoRa frame format, the ToA is derived as follows [58]:

(3) $$\;\;\;\;\;\;\;\;\;\;\;\;\;\;\;\;\;\;\;\;\;\;\;\;\;\;\;\;\;\;\;\;\;\;\;\;\;\;\;\;\;\;\;\;T_{Preamble}=\left(n_{Preamble}+4.25\right)T_{S}$$

(4) $$n_{Data}=8+max\left(ceil\left[\frac{\left(8PL-4SF+28+16CRC-20IH\right)}{4(SF-2DE)}\right]\left(CR+4\right),0\right)$$

where

- $n_{Preamble}$ is 8 by default.
- *PL* is the number of payload bytes (1 to 255).
- *SF* is the spreading factor (6 to 12).
- *IH* = 0 when the header is enabled, IH = 1 when no header is present.
- *DE* = 1 when low data rate optimisation (LDRO) is used, 0 otherwise. LDRO increases the robustness of the transmitted signal. This is particularly beneficial in environments with significant obstacles or long-range communication scenarios.
- *CR* is 1, 2, 3, or 4 for code rates 4/5 to 4/8.
- *CRC* = 1 when the payload CRC is enabled and 0 otherwise.

(5) $$T_{Data}=n_{Data}\cdot T_{S}$$

The transmission time for a LoRa frame, the time on air (ToA), is given by

(6) $$ToA=T_{Preamble}+T_{Data}$$

The overall transmission latency for a frame is given by the sum of the ToA and the propagation delay. However, the propagation delay is significantly smaller than the ToA and hence can be neglected for most purposes. For illustrative purposes, for 30 km, the propagation delay is 100 μs. The ToA for a LoRa frame computed from Equations (1)–(6) with illustrative parameters is given in Table 2.

### 3.2. Range

#### 3.2.1. Average Range

The CSS technique in LoRa increases the range and robustness of the radio communication links compared to traditional modulation techniques. The resulting LoRa receiver sensitivity for the Semtech SX1276 LoRa device is illustrated in Table 2. An increase in the spreading factor increases the receiver sensitivity, which translates to an increase in the communication range.

To estimate the range, we use the log-normal shadowing model [59] to model radio wave propagation. This model is commonly used in wireless communication to account for signal strength variability due to obstacles and environmental features that cause attenuation and random fluctuations. It is particularly useful in complex environments with significant obstacles like buildings, trees, or terrain irregularities. The model describes path loss vs. distance as

(7) $$PL\left(d\right)=PL\left(d_{0}\right)+10\alpha {log}_{10}\left(\frac{d}{d_{0}}\right)+X_{\sigma }$$

where $PL\left(d\right)$ is the path loss at a distance *d* from the transmitter, $PL\left(d_{0}\right)$ is the path loss at a reference distance $d_{0}$, *α* is the propagation exponent, and $X_{\sigma }$ is a random variable having a Gaussian probability density function (PDF) with mean 0 and standard deviation σ. The parameters *α* and σ vary for different environments such as urban, suburban, and mixed or irregular terrain. For the suburban environment chosen for this study, *α* and σ were empirically determined following [59] as 3.3 and 3.5. Also, the path loss at a reference distance of 190 m was determined to be 96 dB. Further details on the empirical estimation of the above parameters are available in Appendix A.

The estimated average ranges for different SFs when the transmit power is 17 dBm are shown in Table 2. 

#### 3.2.2. Effect of Shadowing on Range

However, the average range, as computed above, does not reflect the effects of shadowing. For given values of *α* and σ, we now compute the maximum distance d* for which the received signal power $P_{r}\left(d^{*}\right)$ remains above the receiver sensitivity *S_SF_*, i.e.,

(8) $$\;P_{r}\left(d^{*}\right)=P_{T}-PL\left(d^{*}\right)+G_{R}\geq S_{SF}$$

where $G_{R}$ is the receive antenna gain.

Substituting to (8) from (7) we choose the maximum distance d* such that

(9) $$P_{r}\left(d^{*}\right)=P_{T}-PL\left(d_{0}\right)-10\alpha {log}_{10}\left(\frac{d^{*}}{d_{0}}\right)+X_{\sigma }+G_{R}\geq S_{SF}$$

**Figure 2:**
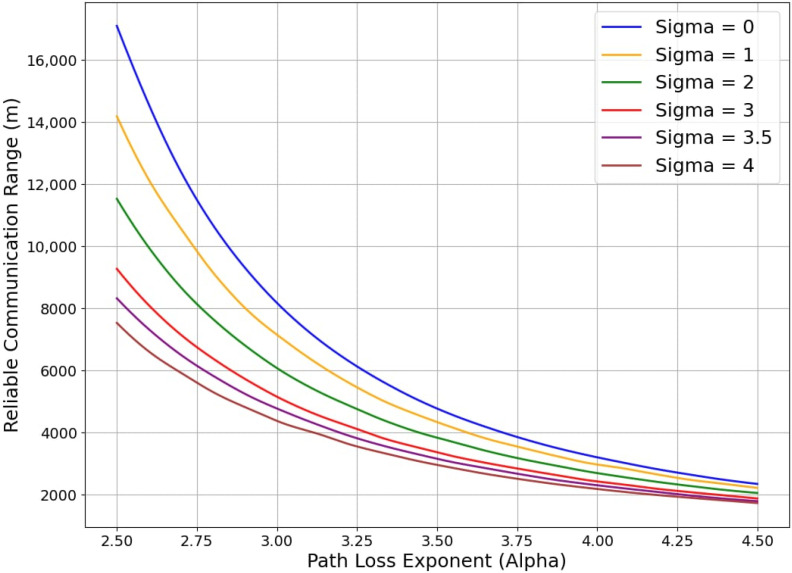
Variation of Reliable Communication Range (RCR) with *α* and σ (SF = 8).

This computation is repeated multiple times with random values for $X_{\sigma }$ taken from an $N\left(0,\;\sigma \right)$ distribution. We then average the values of d* so obtained to estimate the Reliable Communication Range (RCR) for the environment characterised by the chosen *α* and σ. The results of computations using (9) for SF = 8 are illustrated in Figure 2. The number of computations needed to obtain a smooth curve, i.e., to capture sufficient variations in the random component of (9), increases with increasing σ. A total of 250 computations was found to be sufficient for the largest value of sigma shown in Figure 2. Therefore 250 computations were used to obtain all curves in the figure. The transmit power chosen was 17 dBm. It is noted that as σ increases (the environment becomes more and more shadowed), the RCR decreases. For example, for *α* = 3.3, the average range (σ = *0*) is 5.8 km (Table 2). The RCR reduces to 3.5 km when σ is 4. 

#### 3.2.3. Outage Probability

Next, we note that with $X_{\sigma }$ being a Gaussian random variable, there is a finite probability that within the distance $d^{*}$, the received signal power may still fall below $S_{SF}$. We define this as the Outage Probability within an area of radius $d^{*}$, ${Prob}_{out}$. This is the fraction of the area in which the signal strength is below $S_{SF}$. To compute ${Prob}_{out}$, we may proceed as in (10) [60]. We refer to this range analysis in Section 6 when selecting suitable dimensions for the proposed network.

(10) $${Prob}_{out}=\frac{1}{\pi {\left(d^{*}\right)}^{2}}\int_{0}^{d^{*}}2\pi r\left[Prob\;\left\{(P_{r}\left(r\right)<S_{SF}\right\}\;\right]dr$$

### 3.3. Trade-Offs

The examination of Table 2 illustrates how LoRa’s long-range capability is achieved by compromising the data rate and transmission latency. Further, it illustrates the latency advantage of multi-hop communication with LoRa. For the propagation environment and the payload size considered, with SF = 12, a communication average range of 11.7 km is achieved with a latency of 2138 ms. With SF = 8, a range of 5.8 km is achieved with a latency of 175 ms. If the 11.7 km distance is covered with two hops of SF = 8, the latency will be 350 ms (ignoring any processing delay at the intermediate node), approximately 16 th of the latency of a single hop with SF = 12. 

## 4. Proposed Network Architecture

Our decentralised EEW low-cost MEMS ground motion detection sensor nodes installed in the houses of community members are equipped with a LoRa communication interface and act as end nodes (ENs) of the network. When an EN generates an alert message, the proposed LoRa-based network broadcasts it across the network in a multi-hop manner via relay nodes (RNs). RNs are also LoRa devices that may be installed in households or other strategic locations to ensure sufficient connectivity. The goal of this network is to transmit an alert message from its origin to all participating households within a 30 km radius (area of interest) before the arrival of the S-wave. The network leverages the trade-offs between spreading factor (SF), range, and time on air (ToA) to meet this objective.

**Figure 3:**
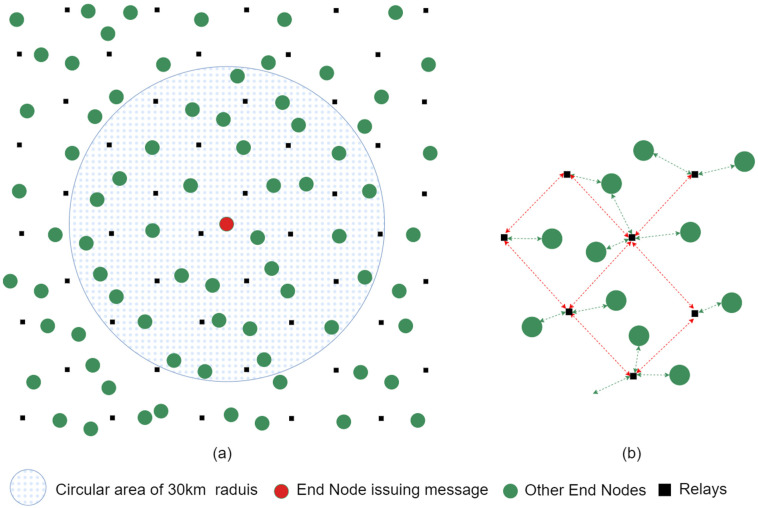
(**a**) Generalised multi-hop broadcast LoRA network architecture. (**b**) Logical network topology. The green arrows indicate RN-EN communication and the red arrows indicate RN-RN communication.

The network architecture proposed in this paper is an application of the multi-hop concept demonstrated in [38] and is illustrated in Figure 3a. An alert generated by an EN in the network is distributed over an area of a radius of 30 km around it via multiple hops, supported by RNs. The ENs form a star topology with each RN within their reach. The broadcast nature of the latter devices creates a mesh among them. Thus, we identify the network architecture as a *mesh of stars*, as illustrated in Figure 3b. All devices in the network operate with a single frequency and a single SF.

Each node transmits as and when it needs to, as per the simple ALOHA-like protocol adopted in LoRa by [29]. However, LoRa’s 1% regulatory duty cycle is enforced [29]. Simple broadcasting, as opposed to routing to a gateway as in LoRaWAN, is adopted. However, to prevent network congestion, a controlled flooding mechanism is adopted to propagate the message over the network. These choices contribute towards the mitigation of network delays. In addition to its location, the transit time of a packet from its origin to a given destination EN (end-to-end latency) predominantly depends on the sum of the ToAs of each hop it traverses and, hence, on the selected SF. The choice of communication parameters and RN positioning is described in Section 6. 

### 4.1. End Nodes (ENs)

**Figure 4:**
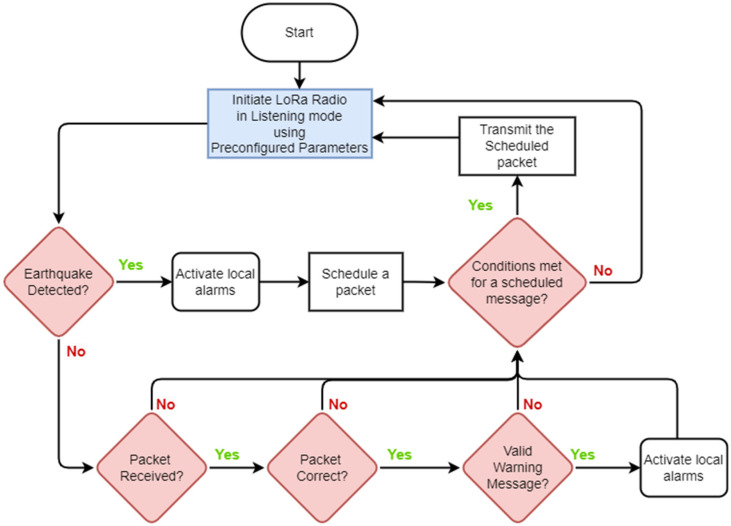
Operation of end nodes.

Figure 4 illustrates the operation of the EN. In the standby mode, the ENs continuously listen to incoming alerts. If the EN verifies that an earthquake has occurred through its ground motion sensor, it activates the local audible/visible alarms and broadcasts an EEW alert message. If the EN receives an EEW alert message over the network, it activates a local alarm. ENs do not repeat (relay) the messages they receive. Scheduling a packet facilitates adhering to the LoRa duty cycle of 1%.

The location and density of ENs are unrestricted, allowing for citizen science (community participation). Existing ENs may become deactivated and new ENs may get activated in different locations at different times. Each EN must be within range of at least one RN. In this work, we assume that ENs are randomly distributed within the area of interest following a uniform distribution. 

### 4.2. Relay Nodes (RNs)

**Figure 5:**
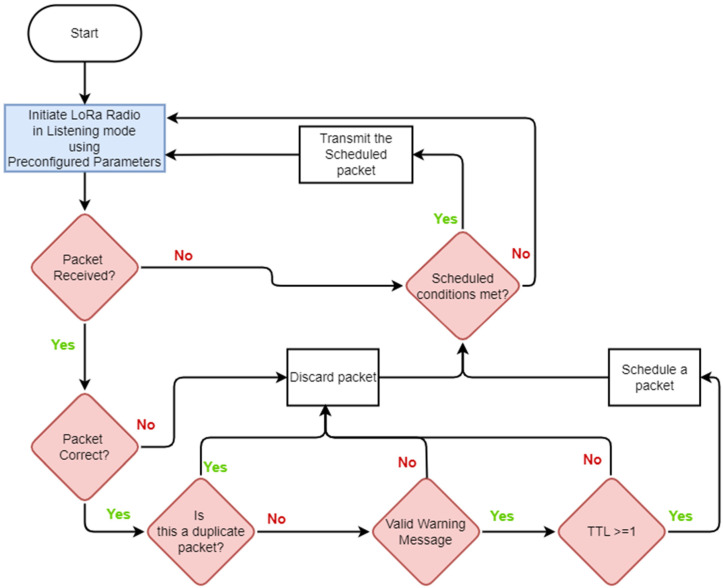
Operation of relay nodes (RNs).

These devices play a critical role in the EEWS. Their primary responsibility is to receive messages from ENs and RNs within their range and broadcast them. Figure 5 illustrates the operations of the RNs.

RNs discard messages that are in error, are duplicates, or whose time-to-live (TTL) counter has expired. RNs may receive duplicates since EEWs can traverse multiple routes. The RN maintains a FIFO cache of the last five messages received to identify duplicates. This is a measure to avoid congestion on the broadcast network. The TTL count prevents messages from being carried beyond the necessary range. To prevent overlap between messages pertaining to different incidents (e.g., EQs), the cache is reset at intervals of one minute. At present, RNs do not have earthquake detection or verification capabilities. 

The placement of RNs is strategically predetermined to ensure optimal coverage of the entire area of interest. This positioning guarantees that all ENs within the system, as well as those that may be added in the future, can reach at least two RNs. Positioning of RNs also considers the need to achieve the lowest possible spatial density (for practical considerations such as cost) and low probability of collision for the selected communication parameters. Table 3 compares the roles and functions of ENs and RNs.

**Figure 6:**
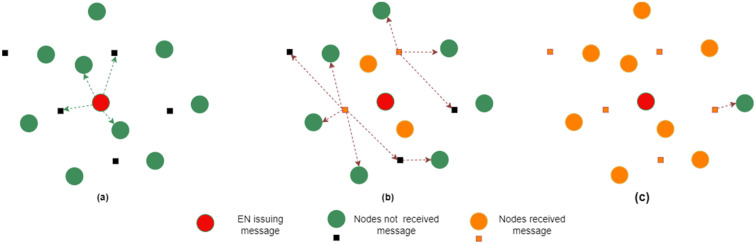
Illustration of disseminating a message through the network. (**a**) Initial broadcast of EEW by EN (**b**) First relay action by neigbhouring RNs (**c**) Second relay action by an RN further away.

Figure 6 shows possible stages in disseminating an EEW throughout the network in a multi-hop broadcast manner.

### 4.3. The Custom Gateway

The Custom Gateway is a reduced-function gateway device with three functions: keep track of incoming messages from the network, initiate health check pings to the network, and initiate parameter configuration of the devices. These housekeeping functions are not central to message dissemination. However, they are crucial to network management. The Custom Gateway uses the Message Queue Telemetry Transport (MQTT) protocol to connect to a network management centre. The RNs and the ENs will receive and respond to Health Check and Parameter Configuration Messages in the same broadcast manner; however, they will have a back-off mechanism to prioritise warning messages. The discussion of these functions is beyond the scope of this paper. The Custom Gateway is a single-channel device and operates on the same LoRa channel and the SF as the RNs and ENs. It uses WiFi to communicate with the central server via the internet. 

### 4.4. Scaled-Down Field Experiments

**Figure 7:**
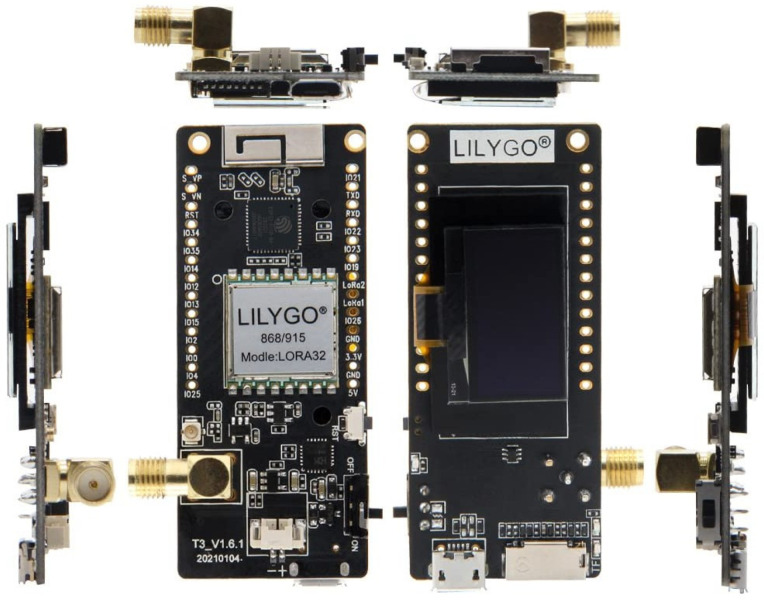
Top (**right**) and bottom (**left**) view of the Lilygo LoRa32 device (Source: [61]).

This section describes a small-scale broadcast LoRa network designed and deployed as a small-scale field experiment of the proposed architecture described above. This exercise is a precursor as a proof of concept at the hardware level to a more detailed simulation study of the architecture. ENs, RNs, and the Custom Gateway are implemented with Lilygo LoRa32 devices [61], as shown in Figure 7, and their key specifications are summarised in Table 4. This device, which is based on the ESP32 microcontroller with Semtech’s SX1276 LoRa transceiver [58], is selected for field testing given its low cost and compatibility with a number of compatible libraries available. A plethora of use cases [62,63,64,65] are found for this device as well. The firmware for the nodes was implemented as per Figure 4 and Figure 5. 

**Figure 8:**
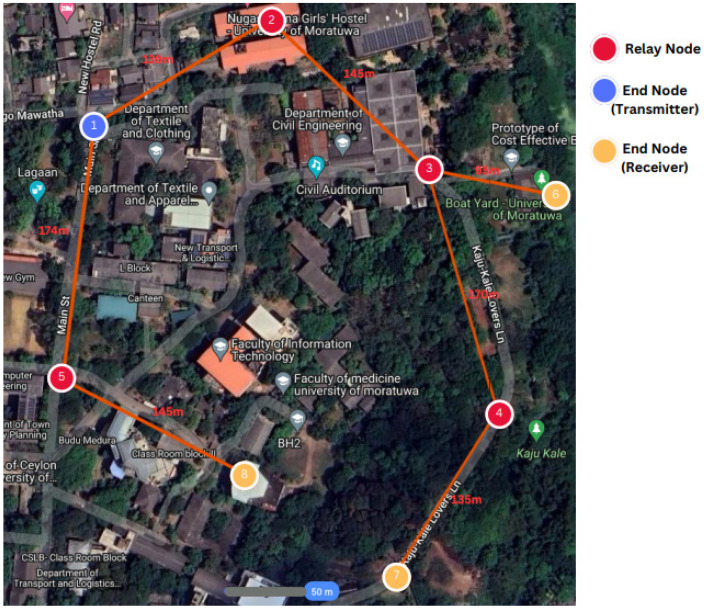
Scaled-down field experiment.

The topology for the scaled-down experiment and the test environment are depicted in Figure 8. The chosen network parameters are listed in Table 5. To limit the size of the network due to practical constraints in experimentation, the transmit power of the devices was set to their minimum level of 2 dBm. An SF of 8 was used. The experimental network consists of four RNs (nodes 2, 3, 4, 5) and four ENs (nodes 1, 6, 7, 8). The test environment presented buildings and trees blocking the line of sight between the nodes. Table 6 shows the height above ground level of the nodes.

Of the ENs, Node 1 was chosen to originate messages, and we observed the reception at ENs 6, 7, and 8. In order to observe routes taken by the alert message, duplicate message suppression was disabled. The observations are summarised in Table 7. Examination of results shows that the relatively higher elevation of Node 2 resulted in good message relay through it. Poor links are identified as 1–3, 4–6, and 5–8 due to practical constraints in placing nodes at higher levels, ensuring lesser shadowing. Overall packet delivery rates of 90%, 82%, and 48% are achieved by nodes 6 (via three routes), 7, and 8, respectively.

This experiment confirms the operation of the multi-hop broadcast network concept and the firmware functionality of the ENs and the RNs. The proposed broadcast LoRa network can relay messages through one or more routes to the receiving ENs. This experiment, though simple, provides a methodology for more detailed experiments to be conducted in order to find better node location via identification of poor links at a given site. It is also a proof of concept to embark on a more detailed simulation study as described in Section 5, Section 6 and Section 7. 

## 5. Network Simulation Platform

### 5.1. The Simulation Tool

Given the impracticality of field experiments with a large network, customising and utilising a suitable simulation environment is important for the design, optimisation, and validation of the proposed network. In this section, we describe the development of a simulation environment to study the proposed network. 

Upon evaluating several simulation tools for LoRa as summarised in Table 8. Framework for LoRa (FLoRa) [53] was identified as the most suitable for our needs. FLoRa utilises the OMNeT++ [66] network simulator with the INET Framework, allowing for the simulation of the LoRaWAN architecture. In this setup, power-constrained end nodes communicate with gateways. Gateways act as intermediaries between the end nodes and the network server, facilitating data exchange. End nodes in FLoRa can only communicate with a gateway using LoRaWAN protocols.

A modified version of FLoRa is presented in [56], exploring the effects of adding multi-hop capability to LoRaWAN. To this end, the authors implement a downlink for LoRa nodes that facilitates direct communication between them without a gateway. We extend this by creating two types of nodes: ENs with bidirectional communication capability and RNs with broadcast capability. The latter capability enables a message to be relayed to all ENs in the network via multiple hops instead of routing to a specific destination. Tools for the analysis and visualisation of the event logs and scalar/vector recordings, entities which are responsible for documenting each state transition, every message transmission and reception, and timestamps were developed along with the simulation tool. With our extended FLoRa simulator [70], we are able to gain insights into message traversal through the network, such as the routes taken by the packets, the number of hops, and the transit time of packets.

### 5.2. The Simulation Model

The simulation tool described above is used in the design of the network and its performance evaluation. This section describes how the message dissemination scenario is modelled within the simulation environment.

**Figure 9:**
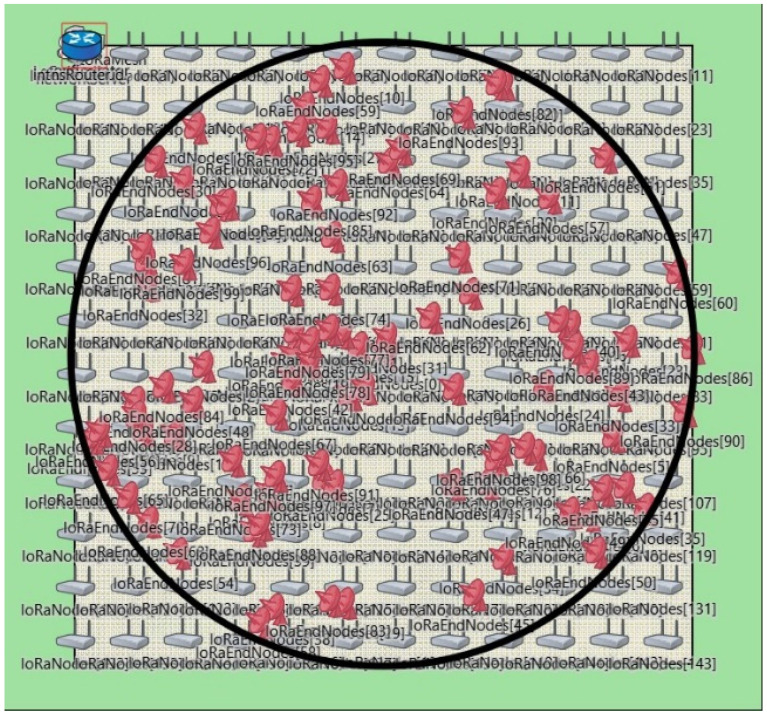
Simulation scenario for network design and evaluation. ENs are depicted in red and RNs in grey. The RN separation and the SF are simulation variables.

Our area of interest is a circular region of a 30 km radius (extracted from a 60 km × 60 km area) centred around the EN generating the EEW. ENs are positioned at random, following a uniform distribution with variable device density. RNs are positioned in a rectangular grid such that each RN is within reach of one or more other RNs. The grid size and the SF are variables to be selected as required. A log-normal shadowing model with its parameters tuned through field experiments, as described in Section 3.2, is adopted for propagation modelling in the simulator. All nodes are single-channel devices and operate with the same SF. The simulation model assumes using the same devices as in the field experiment (Lilygo LoRa32). The receiver sensitivity for each SF is obtained from data sheets of the SX1276 LoRa modulator [58] used in the Lilygo LoRa32. The transmit power for all nodes is 17 dBm, and an antenna gain of 2 dBi is assumed for all devices. The simulation setup for the design and evaluation of the network is shown in Figure 9. 

Each simulation run consists of a single message generated by the EN at the centre of the area of interest. In order to generalise our results, the simulation is run multiple times with different EN positions within the area of interest taken from a uniform distribution. This approach ensures that our network architecture and our results are applicable regardless of the placement of ENs relative to RNs. The results reported in this paper are based on a set of 100 ENs in each simulation run. However, the results are independent of the number of ENs as all ENs except the originating node are receive-only nodes while an EEW message is in transit. 

In Section 6, we describe how this model is used in the selection of the grid size and the SF. We then use this model to evaluate the general performance of the network in Section 7 and then a case study of a specific EEW application in Section 8.

## 6. Network Design

The SF and the spacing of RNs are the key design parameters of the multi-hop network. This section describes how these parameters, which are interdependent, were determined using the extended FLoRa simulator. The section also describes the design of the message structure. 

### 6.1. Grid Size and Spreading Factor

#### 6.1.1. Selection of Grid Size

After some preliminary studies between square, hexagonal, and Voronoi grids, a decision was made to choose the first. The grid size refers to the spacing between RNs. The interrelationships between the SF, the average range, and the range for reliable communication were discussed in Section 3.2. The RCR may be used as a guide to select the RN grid size. Figure 2 (for SF = 8) and similar results for other SFs were used to determine the grid size. As an additional measure to ensure multiple routes through the RN grid, we recommend a slightly more conservative value of 90% of what Figure 2 gives. The chosen grid sizes are shown in Table 9. Since we study the dissemination of a message over the area of interest, we also compute the area covered by a single RN. These values are presented in Table 9. We observed that the Outage Probability computed from (10) was less than 0.12% for these choices, which implies very reliable message delivery.

#### 6.1.2. Selection of Spreading Factor

While fewer RNs and, consequently, a larger SF are desirable, selecting an appropriate SF requires understanding message dissemination latency and collisions. We randomly placed 100 ENs in the target area and ran 25 simulations for each SF, with RN placement as per Table 9. Table 10 shows the results. In some cases, messages did not reach all ENs, primarily due to communication unreliability from shadowing at lower SFs and collisions at higher SFs due to longer ToA. However, multiple routes to the same EN through different RNs have sometimes ensured message delivery to all ENs. As expected, observations show that smaller SFs require more hops but significantly less time to reach all ENs than higher SFs.

From these observations, we found that an SF of 8 is most suitable, as it demonstrates the minimum latency among the SFs that provide 100% coverage of the ENs. Accordingly, from Table 9, we choose a grid size of 3.2 km. We note from Table 9 that for this grid size, an RN covers an area of 10 km^2^. 

### 6.2. Message Structure

**Figure 10:**
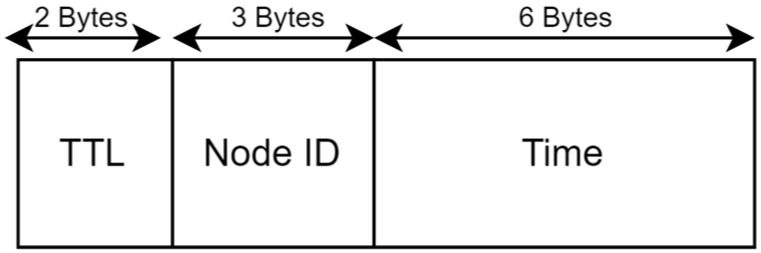
Payload structure of a Warning Message.

The primary focus of this study is the Warning Message. As introduced in Section 4.3, the network also uses Health Check Messages and Network Configuration Messages, which are not described here. Figure 10 shows the payload structure of a Warning Message containing three fields. We use 3 bytes to represent node IDs from 0 to 999 and 2 bytes for the TTL, allowing us to choose up to 99 hops. The detection time in milliseconds is encoded as six hexadecimal digits, making the total message payload 11 bytes. The EQ detection time stamp and the sensor node ID that generated the alert are considered as two primary components of alert data. The message uses $n_{Preamble}=8,$ IH = 0 (header is enabled), CRC = 1 (payload CRC is enabled), DE = 0 (LDRO is not enabled), and CR = 1 (code rate of 4/5 for the payload). Our objective is to illustrate that a small message which helps reduce the ToA suffices to serve as a Warning Message.

## 7. Performance Evaluation

This section presents a detailed performance evaluation of the designed multi-hop broadcast network through simulations. We study the traversal of a generic message through the area of interest. We use the simulation model presented in Section 5.2 with the parameters given in Table 11. We give a ±100 m random displacement to the RNs from the square grid in order to capture realistic constraints when positioning devices. This displacement also contributes to reducing possible collisions between frames arriving at a node via two neighbouring RNs.

### 7.1. Performance Metrics

The key performance metric of the proposed multi-hop broadcast network is the end-to-end latency when disseminating a message. We define the end-to-end latency for each receiving EN as the time elapsed for a message to reach this device since the time it was issued. This is the sum of the processing times at each intermediate RN, the ToA of each hop, and the propagation delay over each hop. Since the ToA takes on values of the order of tens to thousands of milliseconds, it is by far the dominant contributor to the latency. The end-to-end latency varies over the region of interest. Therefore, to obtain detailed insight into the dissemination process, we analyse the following:

- Distance the message travels within the network as a function of time elapsed since its issue. We consider this as the *effective velocity of propagation* of the message through the network.
- Percentage of nodes receiving the message as a function of the time elapsed since its issue. We consider this as the *cumulative probability distribution (CDF) of node penetration* through the network.

In the case study presented in Section 8 specifically for EEW dissemination, we identify further performance metrics.

### 7.2. Results

We examine the performance of several SFs with parameters as shown in Table 8, even though we identified 8 as the most suitable SF in Section 6.1. This helps to confirm our choice. The results presented in this section are for a total of 2500 nodes appearing in the 25 simulation runs for each SF.

#### 7.2.1. Effective Velocity of Message Propagation

**Figure 11:**
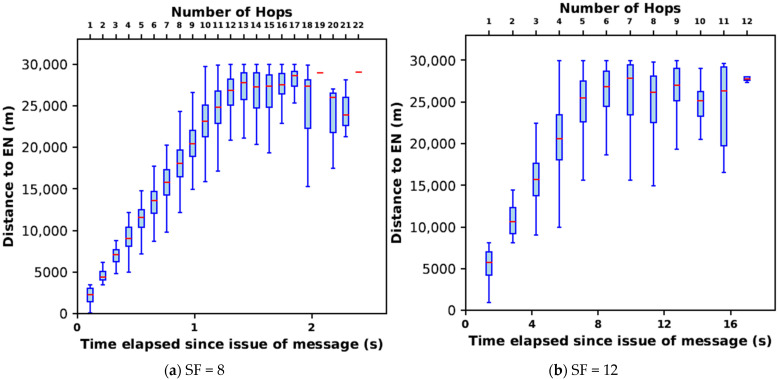
Propagation of a message to ENs within the network.

Figure 11 shows a scatter plot of the distance to each EN vs. time. Each dot represents an EN in the region of interest. For comparison purposes, SF = 8 and SF = 12 are shown in Figure 11a,b, respectively. The alignment of data points in vertical segments illustrates the multi-hop nature of the propagation of messages through the network. Each vertical segment corresponds to one hop. We observe that nodes at different distances receive the message with the same latency and that nodes at approximately the same distance receive the message at different times. This is (1) due to the different message routes in different directions and (2) packets arriving through longer routes (more hops) later while packet losses have occurred over shorter paths. The latter illustrates the presence of multiple routes to an EN. A total of 22 hops are observed for SF = 8 and 12 hops for SF = 12. A linear variation of median distance is seen for hop counts less than 13 for SF = 8 and for hop counts less than 6 for SF = 12. These correspond to message traversal with no loss along the way (shortest route). For ENs towards the edge of the area of interest, more hops are observed, corresponding to longer routes traversed by the messages due to losses along the shortest route. 

For SF = 8, considering the median distances, the effective velocity of message propagation is approximately 20 km/s. Considering the minimum and maximum distances covered for different hop counts, the effective velocities are approximately 4 and 30 km/s. For SF = 8, all except one EN very close to the message origin travel at effective velocities well above the S-wave velocity of 3 km/s [4]. This is despite any packet losses that may have occurred within the network. In contrast, for SF = 12, the median effective message velocity is approximately 3.5 km/s with a large portion of the nodes falling below the 3 km/s mark. From these observations, we conclude that the LoRa-based multi-hop network with SF = 8 has the potential to carry messages through the entire area of interest in a timely manner for EEW before destructive ground shaking occurs. However, the effective message velocity in SF = 12 is inadequate to do so.

#### 7.2.2. CDF of Node Penetration

**Figure 12:**
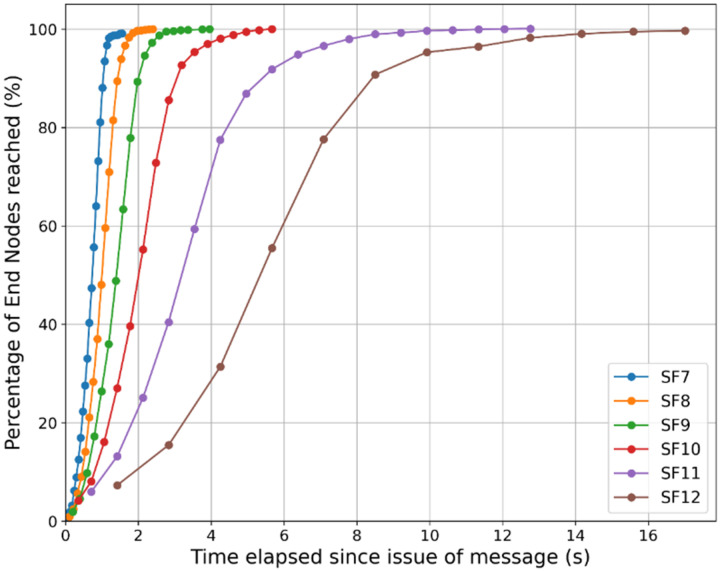
Percentage of end nodes reached vs. time.

The nature of message propagation is further examined in Figure 12, which shows the percentage of ENs reached with time. We observe that the number of hops present in the area of interest for SFs 7 to 12 varies from 26 to 12. With SFs between 8 and 11, the EN penetration is 100%. For SFs 7 and 12 a packet loss of about 1% is observed. The time taken to propagate the message over the area of interest increases from 1.5 s for SF = 7 to 17 for SF = 12. For SF = 8, the value chosen for the multi-hop network in Section 6, 100% penetration is achieved in 2.4 s, despite having to go through up to 22 hops. In this case, 80% of the nodes have received the message within 1.5 s. For SF = 9 and 10, 100% penetration takes 4 s and 5.7 s respectively. The message takes 17 s to cover the area with SF = 12 despite traversing only 12 hops. 

#### 7.2.3. Further Insights and Extension of Results

*Reliability*: The proposed multi-hop network does not attempt to recover lost messages. Messages may be lost due to collisions or propagation anomalies. However, as there are multiple routes to a given destination, as Figure 12 shows, 100% of the ENs receive the message. Message losses manifest as increased latency. The results shown above include the effects of message losses.

*Different propagation environments*: The above sections provided simulation results for an environment characterised by α=3.3 and σ=3.5 and using an SF of 8 in an area of interest containing 100 ENs. To examine the performance for other propagation environments, it is necessary to identify the RN spacing following the process explained in Section 3.2.2 (particularly Figure 2) and Section 6.1. For more severely shadowed environments, it is expected that with SF = 8, the RN spacing would reduce, requiring more hops to cover a given distance and, hence, increased latency. Alternately, we may resort to an SF of 9 with a higher RN spacing and, hence, less latency. The severity of shadowing may be combatted with a higher SF. Conversely, in an environment with less shadowing than the one considered in this paper, an SF of 7 or 6 might be more suitable from a latency standpoint. It is interesting to note from Figure 2 that as σ increases beyond 6, the incremental RN spacing becomes very small. 

*Scalability*: While a message is in transit in the network, only the RNs transmit messages as needed, and the ENs listen and receive. Thus, the proposed system does not have a limitation on the number of ENs that can be supported, though 100 ENs were used in this study for convenience of simulation. 

*Energy*: We exploit LoRa’s low-power feature in our work. We further reduce energy consumption by selecting multiple short hops to cover the area of interest. Further, all the devices in the network are idle though in the switched on state, except when disseminating a message or carrying out housekeeping tasks. In conventional EEWSs, the communication devices are always on, consuming the same power continuously.

## 8. Case Study—Wellington-Based Decentralised EEWS

The performance evaluation presented in Section 7 is for a general scenario of propagating a single message over a 30 km radius area. In order to evaluate the proposed LoRa-based multi-hop, broadcast network as a feasible data transmission technology to be integrated into a real-world EEW solution, we now apply it to our previous experimental performance evaluation of an EEWS consisting of low-cost Raspberry Shake 4D (RS-4D) sensors operated in a decentralised manner with an adapted PLUM-based S-wave-EQ detection algorithm and alert generation driven by node-level data processing as reported in [4]. Originally, the results reported for system latency in our previous study [4] were obtained with the use of the public TCP/IP-based internet backbone for all the data communication needs of the EEWS. In this paper, we replicate our previously reported Wellington-based scenarios on OMNET++ FLoRa-based simulation substituting TCP/IP data transmission with LoRa and compare the results obtained for system latency and the duration of the EEW window. The remainder of this section briefly describes the experimental system, examines the proposed LoRa-based communication architecture as an alternative connectivity means, and compares the two. In this section, we identify all components contributing to the latency within the EEWS. These include the EQ detection time, the S-wave travel dynamics, and the time to verify with a second node before an EEW is issued. We use the selected SF of 8 and the same propagation model as before.

### 8.1. Case Study—Architecture of the Experimental EEWS 

**Figure 13:**
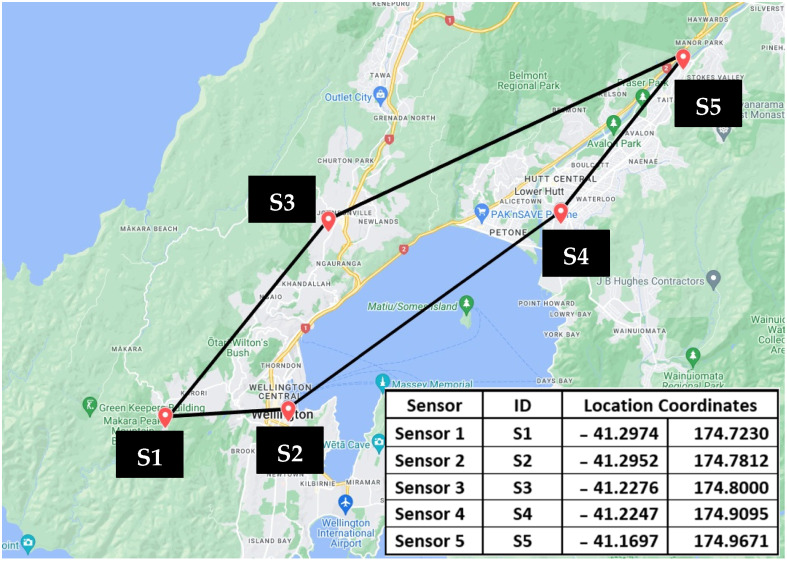
Installed Raspberry Shake sensors in the Wellington region (Source: [4]).

This section describes the experimental EEWS architecture reported in our previously published work [4]. This experiment was conducted in a decentralised sensor network consisting of five RS-4D sensors installed in the homes of community members in the Wellington Region in NZ, as illustrated in Figure 13. This case study used the data obtained from six hypothetical earthquake scenarios to test the performance of the EEWS (please refer to Appendix B for scenario illustrations extracted from [4]). 

In our previous study [4], these earthquake scenarios were used to calculate the S-wave arrival time at each sensor location by considering the EQ epicentre and the direction of the wavefront movement of the S-wave for each hypothetical earthquake. The adapted PLUM-based EQ detection algorithm was used to detect the EQ at each sensor node. In addition to EQ detection, all the sensors are connected to each other via TCP/IP internet-based data communication with installed algorithms required for two stations triggering and verifying warning generation and receipt. The hypothetical scenarios used for the experiments were developed with the sole purpose of evaluating the performance of (1) the PLUM-based EQ detection algorithm, along with the algorithms used for the verified warning generation with two stations triggering, and (2) data communication of the sensor network and system latency. Having considered the physical locations of the five sensors installed, the epicentres of the hypothetical scenarios were defined such that their azimuthal direction and the point of arrival of the S-waves at the sensors could provide the opportunity to observe both lower and higher warning times in accordance with the adapted PLUM approach. Importantly, scenarios are not designed to issue warnings for the end users but only to capture important experimental data to evaluate the several key performance indicators of the EEWS. 

As the first step of the experiment of identifying the system latency for each EQ scenario, the actual triggering time of the algorithm at each of the sensors was obtained by calculating the arrival time of the S-wave at each sensor from the epicentre of the EQ by using the S-wave velocity of 3 km/s. As given in Appendix A, the epicentre of each EQ scenario was predefined. For example, the epicentre of Scenario 1 was defined as −38.89, 178.54. Similarly, the physical location of each of the five sensors installed in the Wellington region is also known (e.g., Sensor 1 is installed at −41.2974, 174.723, see Figure 13 for location of other sensors). Therefore, with the known epicentre and sensor location, the time taken for the S-wave to arrive at each sensor was calculated for each scenario. 

As the next step of system latency calculation for a particular EQ scenario, time-stamped hypothetical ground motion datasets with the calculated S-wave arrival time at each of the Sensors 1–5 were programmed into the EQ data simulator installed within the sensor. For the hypothetical earthquake data, we used a ground motion data set captured by our RS sensor network. This EQ data simulator runs on the RS-4D embedded Raspberry PI computer and was developed [4] to simulate the output of an RS-4D three-axis accelerometer data precisely with the same sampling rate. For each scenario, the latency calculation experiment began by triggering the preprogrammed EQ data simulator in a synchronised manner for each sensor at the exact time when the S-wave of that particular earthquake scenario was expected to reach each sensor. Having received the simulated data inputs, the preprogrammed PLUM approach carries out the detection, verification, and alert generation process. The recorded resultant time-stamped data were used to calculate the system latency figures for each EQ scenario.

We assumed that the intensity of the seismic wave for each hypothetical earthquake scenario was sufficient to reach all five sensors located in the Wellington region. Each earthquake scenario was repeated 10 times, totalling 60 experiments. The variability of latency observations for each repeated scenario was within a range of a few milliseconds. Hence, we present average values for each scenario as in the following discussion. Further details of the EQ scenarios and previous experiments are available in Prasanna et al. (2022) [4].

### 8.2. Earthquake Detection and Verification

**Figure 14:**
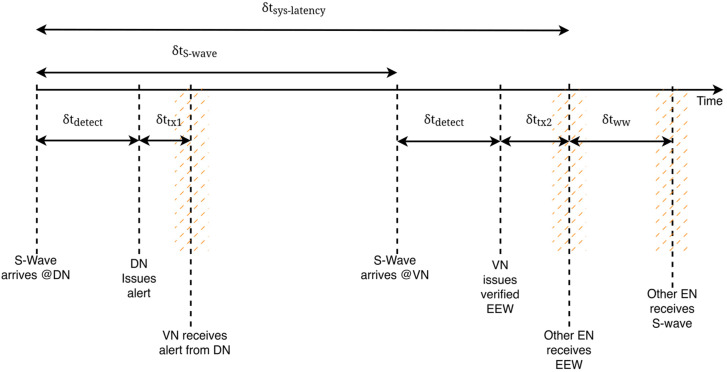
Detecting and verifying an earthquake by sensor nodes.

As mentioned, we adopt a two-stage verification scheme to enhance the reliability of alert generation [4]. A warning is issued only after two nodes sense an earthquake. The detection node (DN) is the node first to receive the S-wave. After executing the EQ detection algorithm, the DN issues an unverified message, which we name as *an alert*. Nearby nodes, which are potential verification nodes (VNs), receive this and begin listening to the arrival of an S-wave. If a potential VN does sense the S-wave within its listening window, it runs its EQ detection algorithm. If a positive detection is made, the alert from the DN is verified, and the VN issues a verified message, which we name as an *EEW*. This is received by all nodes in the system subsequently. If none of the ENs receiving the alert issued by the DN detects an S-wave within its listening window, it is considered to be false and is discarded. This process is illustrated in Figure 14. The hashed sections pertain to the LoRa multi-hop network only and show the variability of timelines due to multi-hopping. The time for a message to reach a given node will depend on its location and the route taken by the message. We analyse the minima and maxima of these timelines later in this section.

### 8.3. System Latency, Warning Window, and Key Contributing Components

In Figure 14, the following time periods are identified:

- ${\delta t}_{detect}$: The time taken to execute the EQ detection algorithm in the nodes. This depends on the type of the algorithm and its complexity, the nature and direction of the S-wave, and also the specifications of the ground motion detection sensor hardware, as described in [4].
- ${\delta t}_{S-wave}$: The time interval between S-wave reception at the DN and the VN.
- ${\delta t}_{tx1}{,\;\delta t}_{tx2}$: DN to VN and VN to other EN transmission times, respectively, in communication via the available internet backbone infrastructure, ${\delta t}_{tx1}={\delta t}_{tx2}={\delta t}_{tx}\;$. During the experiments, we observed that transmission time variability was negligible with TCP/IP connectivity despite the scattered locations of the nodes (as reported in [4]. However, in the LoRa-based multi-hop network, these components will vary depending on the distance between the communicating nodes and the route taken by the message, the number of hops, and the ToA on each hop. ${\delta t}_{tx1}\;{and\;\delta t}_{tx2}$ include the sum of the propagation times and the sum of the ToAs on all hops between the two end-points and will be different for different end-points.
- ${\;\delta t}_{ww}$: the warning window is the interval between the arrival of the EEW and the arrival of the S-wave at a given EN. This is the time available for community members at the node to take protective measures, making it the most critical overall performance measure of the system.

The system latency $\;{\delta t}_{sys-latency}$ is defined as the time between the first arrival of the S-wave to the system and the time at which an EN in the system receives the EEW. As seen in Figure 14, this is given by

(11) $${\delta t}_{sys-latency}{={\delta t}_{S-wave}+\delta t}_{detect}+{\delta t}_{tx2}$$

It should be noted that the scattered nature of the sensor locations for this particular case study led to a situation where, for all six scenarios, ${\delta t}_{S-wave}>$ ${\delta t}_{detect}+{\delta t}_{tx1}$ and hence the applicability of (11) across all six scenarios In this situation, it is noted that ${\delta t}_{detect}$ at the DN and ${\delta t}_{tx1}$ pertaining to DN-VN communication do not contribute to the system latency and have not been reported [4]. 

However, as shown in Table 12, other scenarios of detection and verification may arise where ${\delta t}_{S-wave}<$ ${\delta t}_{detect}+{\delta t}_{tx1}$, i.e., a second node will receive the S-wave before receiving the (unverified) alert issued by the first node. 

In such a situation, there is a possibility that the second node will also issue an (unverified) alert about an oncoming EQ and share it among all the nodes. As a result, a node that detects the EQ first can become both the first DN of the EQ and the VN and, as a result, may first issue the actual verified EEW. This scenario can occur when the node density is high (closely spaced nodes such that ${\delta t}_{S-wave}$ is small), the detection algorithms are comparatively slower (high ${\delta t}_{detect})$, or communication between nodes is slow (longer ${\delta t}_{tx1}$ and ${\delta t}_{tx2})$. In addition, in a rare occurrence, there is a possibility that a VN can obtain two unverified alerts from two different sensors prior to detecting an EQ. In the case of using LoRa for communication, this is a particularly important consideration when benchmarking or comparing, especially as the multi-hop LoRa transmissions proposed in this paper will take higher ${\delta t}_{tx1}\;$ and ${\delta t}_{tx2}\;$ compared to internet-based TCP/IP transmissions. We will investigate this in detail subsequently in this section.

The results of system latency (${\delta t}_{sys-latency})$ from the case study experiments are summarised in Table 13, including the roles of the nodes. ${\delta t}_{sys-latency}$ varied across the six EQ scenarios. In particular, ${\delta t}_{detect}$ for the same VN varied in different scenarios as well as ${\delta t}_{S-wave}$ between the DN and VN. These are dependent on the algorithm and performance of the sensor node that processes the EQ detection and the direction of the S-wave and the node distribution in the network. Our previous publication [4] describes in detail how measurements were performed in the experimental evaluation of these components. Table 1 also confirms that ${\delta t}_{S-wave}>$ ${\delta t}_{detect}+{\delta t}_{tx1}$ by a significant margin.

### 8.4. Comparison of Findings with LoRa Multi-Hop Network

**Figure 15:**
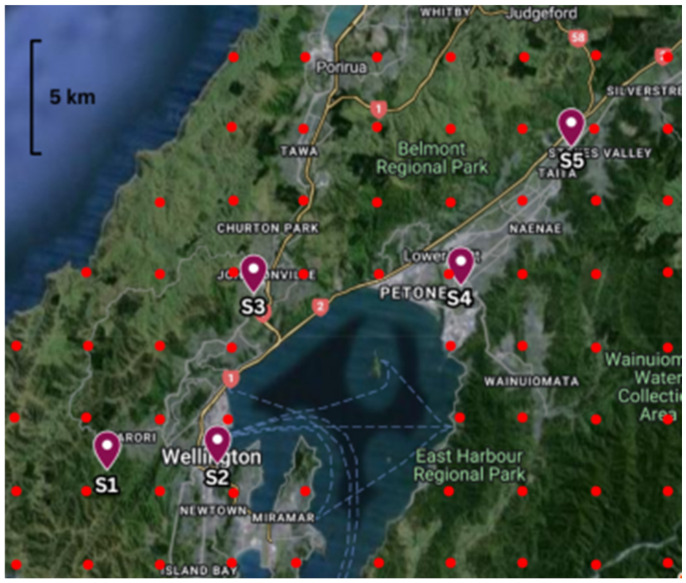
Map of the EQ sensors (ENs) and the RN overlay (shown in red).

In this section, we apply the LoRa-based multi-hop broadcast network designed in Section 6 as substitute technology to replace its original method of data communication between the sensors in the specific case study presented above. Our objective is to compare its performance with the previous TCP/IP-based system. Each sensor is assumed to be an EN equipped with a LoRa transceiver. Due to the difficulty of establishing a physical network, we resort to a simulation study. We use our extended FLoRA simulation tool presented in Section 5.1 to position end nodes (ENs) S1 to S5 at the same locations as in the experiment reported in [4]. We then create an overlay of relay nodes (RNs) on the area. An SF of 8 is adopted, and the grid size of the RNs is chosen accordingly (3.2 km), with a ±100 m random offset given to each RN. No RNs are placed in the area covered by the ocean. The map of the case study simulation environment is shown in Figure 15. Further, in order to ensure generalisation of results, in each EQ scenario, we carry out 25 simulations with randomly selected rotations and displacements of the RN grid relative to the nodes. The propagation model used is the same as before.

As the latency components ${\delta t}_{tx1}$ and ${\delta t}_{tx2}$ only are affected by the change in connectivity from TCP/IP to LoRa, we study these latencies as follows:

- ${\delta t}_{tx1}$ (transmission of an alert from DN to VN): Across the six scenarios, there are only the two DN-VN pairs, S5(DN)->S4(VN) and S1(DN)->S2(VN), that need to be investigated for ${\delta t}_{tx1}$. We generate a message from each of the DNs and observe its travel over the network to reach the corresponding VN.
- ${\delta t}_{tx2}$ (transmission of the EEW from VN to all other nodes): For Scenarios 1, 2, and 6, we investigate ${\delta t}_{tx2}$ for the multi-hop links between S4 and each of S1, S2, S3, and S5. For Scenarios 3, 4, and 5, we investigate ${\delta t}_{tx2}$ for the multi-hop links between S2 and each of S1, S3, S4, and S5. We generate a message from each of the VNs and observe its travel over the network to reach the destination nodes.

We adopt the following three-step procedure:

- Step 1: verify whether ${\delta t}_{S-wave}>$ ${\delta t}_{detect}+{\delta t}_{tx1}$ holds for LoRa multi-hop communication.
- Step 2: analyse ${\delta t}_{sys-latency}$ and compare it with the TCP/IP-based system.
- Step 3: analyse ${\delta t}_{ww}$ and compare it with the TCP/IP-based system.

**Figure 16:**
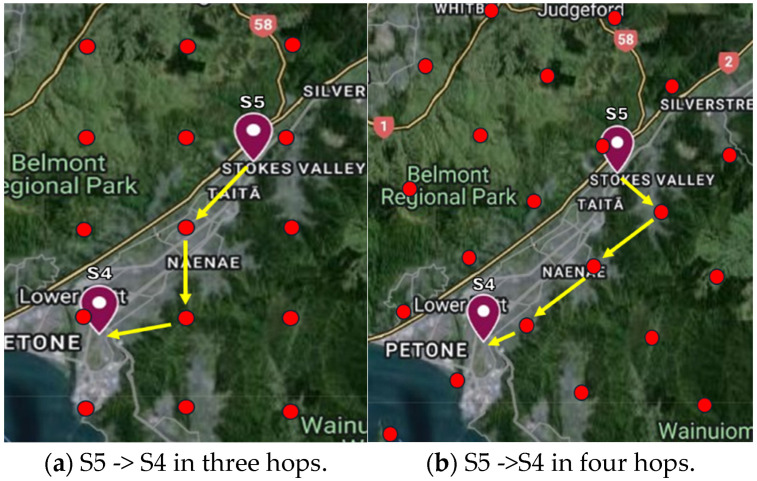
S5 -> S4 message propagation for two instances of RN grid placement (lowest-latency routes).

Step 1:

As explained above, ${\delta t}_{tx1}$ and ${\delta t}_{tx2}$ are expected to be higher in the proposed LoRa-based multi-hop approach than on the internet-based approach, as used in the original case study. Thus, it is important to first check whether S2 and S4 can still act as the EQ verifier/EEW generator, i.e., if the condition ${\delta t}_{S-wave}>$ ${\delta t}_{detect}+{\delta t}_{tx1}$ still holds. In order to verify this, the range of values observed for ${\delta t}_{tx1}$ in all 25 simulations are summarised in Table 14 (Findings of all the 25 simulations for each case are attached in Appendix C). Simulation findings indicate that transmission from S1 to S2 takes two to three hops, while, primarily due to the comparatively larger distance between the two nodes, transmission from S5 to S4 takes three to four hops. The traversed distance also shows differences due to the message taking different routes in different simulation runs. As an illustration, Figure 16 shows two message propagation scenarios for S5 to S4 for two different RN grid placements. The routes shown are those of lowest latency for each choice of grid. Examination of Figure 16 demonstrates the availability of multiple alternate routes as well. Figure 16a shows that if the three-hop route fails, there are two four-hop routes that can provide redundancy within the latency limits computed later in this paper. Similarly, Figure 16b shows that in addition to the identified four-hop route, there are several other routes of the same length.

The maximum ${\delta t}_{tx1}$ times taken for DN -> VN transmission, being 0.436 s and 0.327 s, are significantly higher than the 0.05 s observed with the TCP/IP network (See Table 12 earlier). Though not reported in the original case study, we did observe that our measurements for ${\delta t}_{detect}$ at S1 and S5 varied in the range of 0.09 s–0.17 s. To verify the condition ${\delta t}_{S-wave}>$ ${\delta t}_{detect}+{\delta t}_{tx1}$, Table 15 below tabulates the worst-case scenarios. It shows that even with the LoRa-based multi-hop network, the above condition holds for the studied scenarios. Hence, S4 and S2 act as VNs as in the case of the TCP/IP network. Therefore, Equation (11) still applies to the system latency for the LoRa network as well.

Step 2:

We next examine the system latency with LoRa-based communication. We report the transmission results (${\delta t}_{tx2})$ obtained from 25 simulations for the EEW generated at S2 and S4 in Appendix D and Appendix E respectively. We observe that the EEW traversed over as many as 12 hops in some instances. From the simulation results, we use only the corresponding maximum and minimum ${\delta t}_{tx2}\;$ for all the scenarios for transmitting the warning generated at both S2 and S4 to calculate the corresponding system latency figures. Therefore, by using Equation (11) in Table 16, we report the maximum and minimum values for ${\delta t}_{sys-latency}$ for the LoRa-based EEW. 

It should be noted that except for substituting TCP/IP-based transmission times (*δt_tx2_*) with the LoRa-based maximum and minimum obtained from Table 13 and Table 14, both *δt_s-wave_* and *δt_detect_* remain the same as the figures presented for the original case study in Table 12.

Step 3:

Having obtained the system latencies for all six scenarios with warnings originating either at sensor 2 or 4, we compare the potential total warning window (${\;\delta t}_{ww}$) expected at each recipient node with communication approaches of LoRa-based versus internet-based TCP/IP below in Table 17. From the data obtained from the original case study [4], for all the EQ scenarios, the ${\;\delta t}_{ww}$ for each sensor was calculated based on the S-wave travel time between the epicentre of the EQ and the sensors. For the LoRa-based calculations, we reported the minimum and maximum ${\;\delta t}_{ww}$ calculated based on the min–max latencies reported in Table 15.

From the summary findings of the 25 simulations conducted for LoRa-based communication reported in Table 15, we confirm that the proposed multi-hop broadcast network can transmit EEWs between community-based nodes within a 30 km radius. As shown in Table 17, the spread of the warning window (${\;\delta t}_{ww}$) with LoRa-based communication varied from a minimum of 1.01 s to a maximum of 5.93 s. Importantly, we observed that the minimum ${\;\delta t}_{ww}$ of 1.01 s was reported when LoRa transmission occurred with three hops but can be increased considerably to reach 1.21 s when transmission occurred with two hops. In comparison, the corresponding ${\;\delta t}_{ww}$ spread from internet-based data communication was between 1.51 s and 6.75 s. Therefore, despite trailing behind TCP/IP-based data communication, the findings reported in Table 17 provide clear evidence to confirm that the LoRa-based multi-hop approach demonstrates comparable performance to the public TCP/IP infrastructure as a feasible communication technology for EEW if and when needed. Annexure F reiterates the above quantitative comparison graphically and illustrates the close trailing of LoRa performance against TCP/IP data communications. 

## 9. Discussion and Conclusions

LoRa has become a top choice for transmitting small amounts of data over long distances and providing connectivity where internet infrastructure is lacking. Its capabilities have led to many applications in areas like rural farming and smart metering, where small but important data packets are sent to central servers for processing and decision making.

In disaster contexts, LoRa is rarely used as the sole option for long-range communication. In those rare occasions, almost in all the occasions, LoRa was considered for post-disaster scenarios, with throughput being a priority rather than transmission latency. However, there are very limited known applications where LoRa’s feasibility has been tested in scenarios where (1) low latency is crucial and (2) no internet service is available. Decision making when responding to emergencies is complex and particularly challenging for fast-moving catastrophic hazards like EQs. The speed of information matters most in such situations, as life-saving decisions can become obsolete in a matter of seconds. 

Recognising the research gap and the need for reliable communication when the internet may be unavailable after a major earthquake, in this study we address the question:

“Can a LoRa-based multi-hop communication approach support reaching latency levels required for EEW systems with decentralised EQ detection and support uninterrupted service”.

### 9.1. Key Achievements

As explained very clearly in the findings, this study managed to demonstrate the feasibility of LoRa as the primary data transmission technology for EEWs. This was clearly demonstrated by achieving system latencies low enough to surpass the speed of travel of destructive ground shaking. This is achieved by implementing a multi-hop broadcast architecture on the LoRa physical layer to successfully disseminate EEWs generated by community-based decentralised MEMS sensors using PLUM-based S-wave detection. Through simulations, we show that our network is capable of relaying messages to receivers within a 30 km distance in 2.4 s or less, using up to 22 hops. We further observe the presence of multiple routes for messages within the network, which enhances reliability. A message that may get lost or corrupted over a shorter route may still be delivered over a longer route, albeit with a longer delay. Further, while a message is in transit, it is received via all the ENs in the vicinity. Therefore, the system is scalable to any number of ENs. 

To validate, we established the feasibility of our findings in the real-world context through field experiments. Applying this LoRa-based architecture to facilitate communication for five MEMS-based EQ sensors installed in the Greater Wellington area in NZ, we demonstrate warning windows that are 1.01 to 5.93 s ahead of an earthquake. Though slightly slower than the internet-based systems, the study findings clearly demonstrate that LoRa-based multi-hop communication can achieve a meaningful warning window for decentralised EEWSs, particularly for aftershock sequences, until mainstream telecommunication infrastructure is restored. While these warning windows may seem short, they allow uninterrupted EEW service for the public to prepare psychologically for an impending EQ and take simple protective measures like “drop, cover, and hold.” They are also most sufficient for automated actions such as turning off or slowing down machinery. 

### 9.2. Limitations and Challenges

In this study, we assume that the propagation model parameters determined in our field experiments at the University of Moratuwa are, in an average sense, applicable to the Greater Wellington region in NZ, where the system for our case study is deployed. Though the two areas have similarities in their suburban nature, the terrain differs, resulting in different favourability (both positive and negative) for radio wave propagation. The propagation environment in the Wellington area or any other area in which the system is to be deployed must be characterised empirically, e.g., as in [59,71], and the network design process described in Section 6 must be followed for more accurate results on system latencies and warning time. 

The main challenges in deploying the proposed network on a large scale lie in the positioning of RNs. A single propagation model may not be capable of representing a geographically diverse terrain. However, guidelines provided in Section 3.2.2 and 6.1 may be utilised, together with field trials to establish a successful network. Other challenges include powering and maintenance of the devices if they are not installed in households (e.g., in sparsely populated areas).

### 9.3. Further Work

Our results show the network’s end-to-end performance (from the EN issuing the message to the other ENs receiving it). However, further insights into message passage, collisions, packet losses, and the tolerance of the network to failure of RNs are required to optimise the design further. In particular, the identification of RNs that are involved in the lowest-latency route, those that may be critical, and those that can be used as backups while staying within the latency limits are useful insights. 

While we studied the propagation of a single message launched into the network, it is possible that multiple nodes will detect an EQ and issue messages around the same time. The prevention of this, or the behaviour of the network in response to such near-simultaneous launch of messages, is identified as an important extension of this work. While we exploit LoRa’s long-range capabilities, the energy aspects of the network remain to be investigated. This is particularly important since our ENs and RNs have to be always switched on and listening. Security aspects are also yet to be addressed.

In this study, the RNs’ role is only to broadcast messages over multiple hops. The system would be more attractive if the ENs had relaying capability as well instead of having dedicated RNs. This would be the ideal situation in a dense network of community-based EQ sensors. However, having a dense network of relaying nodes would be detrimental in the sense of collisions. Therefore, in a dense network, identified ENs only could perform the dual role of relaying.

Though we primarily compare our work with EEWs with internet-based connectivity, a significant set of early warning systems based on mobile technologies have seen significant advances [72]. We intend to investigate the performance of our system against these as well. 

More recently, our ongoing parallel research on EEW with the PLUM algorithm has shown success in P-wave-based EQ detection [73]. This offers the potential to increase the warning window for the LoRa-based approach, making its use more feasible and meaningful.

As an initial study, we base our work on low-cost, simple, single-channel LoRa devices. Further investigation is warranted on multichannel operation as well as using higher channel bandwidths.

Furthermore, having demonstrated its capability for applications requiring latencies of a few seconds, it can be argued that with appropriate adaptations, the multi-hop approach in this study can become a more general data communication platform useful for a number of other use cases during and immediately after a disaster. Such applications may include emergency alerting, I’m Safe messages, and building and infrastructure health checks.

## Figures and Tables

**Table 1:** Comparison of extreme physical layer settings in LoRa.

	A: LoRaWAN Recommended Setting (for LPWAN Applications)	B: Fastest Data Rate Setting
SF	12	6
BW (kHz)	125	500
Code rate	4/5	4/5
Range	High	Low
Reliability	High	Low
Energy Consumption	High	Low
Data rate/latency	Low/high	High/Low

**Table 2:** Illustrative values for ToA and range for different SFs.

SF	Receiver Sensitivity (dBm) * [58]	ToA (ms) **	Estimated Average Range (km) ***
6	−118	56.448	3.3
7	−123	97.536	4.7
8	−126	174.592	5.8
9	−129	328.704	7.1
10	−132	616.448	8.8
11	−133	1150.976	9.5
12	−136	2138.112	11.7

* BW = 125 kHz; ** BW = 125 kHz, PL = 50 bytes, $n_{peramble}=8\;$ bytes, IH = 0, CRC = 1, CR = 1, DE = 0; *** propagation model used: log-normal shadowing (*d*0 = 190 m, *σ* = 3.5, *α* = 3.3, *PL*(*d*0)(*dB*) = 96), P_T_ = 17 dBm, G_R_ = 2 dBi.

**Table 3:** Comparison of end nodes and relay nodes.

Feature	End Nodes	Relay Nodes
Operation mode	Always on	Always on
Device density	Can vary over time	Fixed
Device placement	Unrestricted	Predetermined
Role	Sensor, transceiver, alarm	Repeater (broadcast mode)

**Table 4:** Key specifications of the Lilygo LoRa32 device.

Specification Lilygo LoRa32 Device	Description
Microcontroller	ESP32
Operating frequency (MHz)	868, 915, 923
LoRa Chip	SX1276
Tx power	2–17 dBm and 20 dBm
Antenna gain	2 dBi
Wireless protocol	Wi-Fi + Bluetooth 4.2

**Table 5:** The configuration of the field experiment.

Parameter	Value/Description
ENs	Nodes 1, 6, 7, 8
RNs	Nodes 2, 3, 4, 5
Transmit power	2 dBm
Receive antenna gain	2 dBi
Channel	923 MHz
Bandwidth	125 kHz
Spreading factor	8
Payload size	11 bytes
Number of test messages transmitted	100

**Table 6:** Node placement in the field experiment.

Node	Estimated Height above Reference Level * (cm)	Remarks
1 (EN/transmit)	700	Elevated ground level
2 (RN)	1000	First floor of a residential building
3 (RN)	200	Hand-held node
4 (RN)	200	Hand-held node
5 (RN)	800	Elevated ground level
6 (EN/receive)	80	Node set up on a boat
7 (EN/receive)	120	Hand-held node
8 (EN/receive)	1500	First floor of an auditorium

* Water level in the university boat yard.

**Table 7:** Observations of the field experiment.

Originating EN	Intermediate RNs	Receiving EN	Observations	Packet Delivery Ratio (%)
1	3	6	2 hops	28
1	2–3	6	3 hops	61
1	2–3-4	6	4 hops	1
1	2–3-4	7	4 hops	82
1	5	8	2 hops	48

**Table 8:** Comparison of key features among leading simulation tools for LoRa (Source: [67]).

Features	NS-3 [68]	LoRaSim [69]	FLoRa
License type	Open source	Open source	Open source
Operating system	Linux, Windows	Linux, Windows, MacOS	Linux, Windows, MacOS
Type language	C++, Python	Python	C++
Installation requirements	Import all libraries online	Simpy, Numpy, Matplotlib	OMNeT++ INET
GUI	Yes but not for LoRa	Only plot	Yes
Community support	Very good	Limited	Limited
Last update	October 2020	2020	November 2020
Last version	ns-3.32	10 July 2017 n/a	6.0
Popularity	High	High	Medium

**Table 9:** Relay node separation (grid size) for different spreading factors.

Spreading Factor	Computed RN Spacing (m)	Selected RN Spacing ** (m) d*	Number of Relay Nodes (for 60 km × 60 km Area)	Relay Node Coverage (km^2^)
7	2929	2600	529	6.8
8	3524	3200	361	10.0
9	4272	3800	256	14.0
10	5358	4800	169	21.3
11	5826	5200	144	25
12	7178	6500	100	36

** Propagation model used: log-normal shadowing (d0 = 190 m, σ = 3.5, α = 3.3, PL(d0) (dB) = 96) and GR = 2 dBi.

**Table 10:** Message dissemination statistics within the area of interest for different spreading factors.

SF	Avg. Unreached ENs	Avg. Unreached RNs	Max. Time Taken to Reach All Nodes	Avg. Time Taken to Reach All Nodes	Max. Number of Hops Observed
7	0.92%	10.05%	1.5 s	0.86 s	23
8	0.00%	0.34%	2.5 s	1.18 s	19
9	0.00%	0.00%	4 s	1.75 s	17
10	0.00%	0.06%	6 s	2.46 s	14
11	0.04%	0.20%	12.5 s	4.22 s	13
12	0.36%	0.38%	17.5 s	5.69 s	11

**Table 11:** Simulation parameters.

Parameter	Value	Parameter	Value
RF channel	923 MHz	$d_{0}$	190 m
Spreading factor	8 (studies 7–12 as well)	$P_{L}(d_{0})$	96 dBm
Channel bandwidth	125 kHz	α	3.3
Code rate	4/5	σ	3.5
Transmit power	17 dBm	Payload size	11 bytes
Receiver sensitivity	−126 dBm	Number of ENs	100
Receive antenna gain	2 dB	RN spacing	d* as in Table 8 ± 100 m

**Table 12:** Possible detection and verification scenarios at a sensor node.

Possible Detection and Verification Scenarios	Output
First received an unverified alert, and afterwards, EQ was detected.	Verified alert
First detected an EQ and afterwards received an unverified alert.	Verified alert
First received an unverified alert and afterwards received a second unverified alert from a different sensor.	Verified alert
First received an unverified alert, and after waiting for a predefined time window, no further receipt of unverified alerts or detection of EQ (false EQ detection).	No alert
First detected an EQ and afterwards received a verified alert (missed EQ detection).	No alert
Verified alert received before arrival of an EQ.	EQ warning

**Table 13:** System latency with internet connectivity.

Hypothetical Scenarios *	Detecting Node (DN)	Verifying Node (VN) (Issues EEW)	Decentralised Processing Using Mainstream Internet Backbone over TCP/IP (in Seconds)			
			${\delta t}_{detect}$@ the VN	${\delta t}_{tx2}$	${\delta t}_{S-wave}$	${\delta t}_{sys-latency}$
Scenario 1	S5	S4	0.10	0.05	S5->S4—2.50	2.65
Scenario 2	S5	S4	0.13	0.05	S5->S4—2.70	2.88
Scenario 3	S1	S2	0.19	0.05	S1->S2—1.00	1.24
Scenario 4	S1	S2	0.17	0.05	S1->S2—1.20	1.42
Scenario 5	S1	S2	0.17	0.05	S1->S2—1.10	1.32
Scenario 6	S5	S4	0.19	0.05	S5->S4—1.60	1.84

* See Appendix A for illustrations of the scenarios with corresponding azimuthal directions.

**Table 14:** Range of values observed for ${\delta t}_{tx1}$ in all 25 simulations.

Message Transmission	Distance Traversed by the Message (m)		Number of Hops		$\mathrm{Total}\;\mathrm{Delay}\;\mathrm{from}\;S5\;\mathrm{to}\;S4\;({\delta t}_{tx1})(s)$	
	Min	Max	Min	Max	Min	Max
Earthquake verification and EEW generation at S2 (Scenarios 3, 4, 5)						
S1 (DN) -> S2 (VN)	3539	6309	2	3	0.210	0.327
Earthquake verification and EEW generation at S4 (Scenarios 1, 2, 6)						
S5 (DN) -> S4 (VN)	6588	10,591	3	4	0.327	0.436

**Table 15:** Worst-case latency scenarios.

DN -> VN	Minimum Observed ${\delta t}_{S-wave}$ (s)	Maximum Observed ${\delta t}_{detect}$ (s)	Max ${\delta t}_{tx1}$ (s)	Max $({\delta t}_{detect}+{\delta t}_{tx1}$) (s)
S5 -> S4	1.6	0.17	0.436	0.606
S1 -> S2	1.0	0.17	0.327	0.497

**Table 16:** System latency with LoRa-based data communication for EEW originating at S2 and S4.

Earthquake Verification and EEW Generation at S4						Earthquake Verification and EEW Generation at S2					
		*δt_detect_* (s)	*δt_tx2_* (s)	*δt_s-wave_* (s) (S5 -> S4)	*δt_sys_latency_* (s) (LoRa)			*δt_detect_* (s)	*δt_tx2_* (s)	*δt_s-wave_* (s) (S1 -> S2)	*δt_sys_latency_* (s) (LoRa)
**Earthquake Scenario 1**						**Earthquake Scenario 3**					
S4 -> S1	δt_tx2(min)_	0.10	0.982	2.50	3.58	S2 -> S3	δt_tx2(min)_	0.19	0.327	1.00	1.52
	δt_tx2(max)_		0.763		3.36		δt_tx2(max)_		0.545		1.74
S4 -> S2	δt_tx2(min)_	0.10	0.982	2.50	3.58	S2 -> S4	δt_tx2(min)_	0.19	0.654	1.00	1.84
	δt_tx2(max)_		0.654		3.25		δt_tx2(max)_		1.091		2.28
S4 -> S3	δt_tx2(min)_	0.10	0.545	2.50	3.15	S2 -> S5	δt_tx2(min)_	0.19	0.873	1.00	2.06
	δt_tx2(max)_		0.327		2.93		δt_tx2(max)_		1.309		2.50
**Earthquake Scenario 2**						**Earthquake Scenario 4**					
S4 -> S1	δt_tx2(min)_	0.13	0.982	2.70	3.81	S2 -> S3	δt_tx2(min)_	0.17	0.327	1.20	1.70
	δt_tx2(max)_		0.763		3.59		δt_tx2(max)_		0.545		1.92
S4 -> S2	δt_tx2(min)_	0.13	0.982	2.70	3.81	S2 -> S4	δt_tx2(min)_	0.17	0.654	1.20	2.02
	δt_tx2(max)_		0.654		3.48		δt_tx2(max)_		1.091		2.46
S4 -> S3	δt_tx2(min)_	0.13	0.545	2.70	3.38	S2 -> S5	δt_tx2(min)_	0.17	0.873	1.20	2.24
	δt_tx2(max)_		0.327		3.16		δt_tx2(max)_		1.309		2.68
**Earthquake Scenario 6**						**Earthquake Scenario 5**					
S4 -> S1	δt_tx2(min)_	0.19	0.982	1.60	2.77	S2 -> S3	δt_tx2(min)_	0.17	0.327	1.10	1.60
	δt_tx2(max)_		0.763		2.55		δt_tx2(max)_		0.545		1.82
S4 -> S2	δt_tx2(min)_	0.19	0.982	1.60	2.77	S2 -> S4	δt_tx2(min)_	0.17	0.654	1.10	1.92
	δt_tx2(max)_		0.654		2.44		δt_tx2(max)_		1.091		2.36
S4 -> S3	δt_tx2(min)_	0.19	0.545	1.60	2.34	S2 -> S5	δt_tx2(min)_	0.17	0.873	1.10	2.14
	δt_tx2(max)_		0.327		2.12		δt_tx2(max)_		1.309		2.58

**Table 17:** Comparison of warning window (${\delta t}_{ww}$) available with internet-based vs. LoRa-based data communication for EEW generated at S2 and S4.

Earthquake Verification and EEW Generation at S4							Earthquake Verification and EEW Generation at S2						
		*δt_sys_latency_* (s) (LoRa)	*δt_sys_latency_* (s) (TCP/IP)	*δt_s-wave(S)_* S1 -> Sn (n=S1-S3)	δt*_ww_*(s) (LoRa)	δt*_ww_*(s) (TCP/IP)			*δt_sys_latency_* (s) (LoRa)	*δt_sys_latency_* (s) (TCP/IP)	*δt_s-wave(S)_* S1 -> Sn (n=S3-S5)	δt*_ww_*(s) (LoRa)	δt*_ww_*(s) (TCP/IP)
**Earthquake Scenario 1**							**Earthquake Scenario 3**						
**S4 -> S1**	**δt_tx2(min)_**	**3.58**	**2.65**	**8.27**	**4.69**	**5.62**	**S2 -> S3**	**δt_tx2(min)_**	**1.52**	**1.24**	**3.34**	**1.82**	**2.10**
	**δt_tx2(max)_**	**3.36**			**4.91**			**δt_tx2(max)_**	**1.74**			**1.60**	
**S4 -> S2**	**δt_tx2(min)_**	**3.58**		**6.95**	**3.37**	**4.30**	**S2 -> S4**	**δt_tx2(min)_**	**1.84**		**5.11**	**3.27**	**3.87**
	**δt_tx2(max)_**	**3.25**			**3.70**			**δt_tx2(max)_**	**2.28**			**2.83**	
**S4 -> S3**	**δt_tx2(min)_**	**3.15**		**4.99**	**1.84**	**2.34**	**S2 -> S5**	**δt_tx2(min)_**	**2.06**		**7.77**	**5.71**	**6.53**
	**δt_tx2(max)_**	**2.93**			**2.06**			**δt_tx2(max)_**	**2.50**			**5.27**	
**Earthquake Scenario 2**							**Earthquake Scenario 4**						
**S4 -> S1**	**δt_tx2(min)_**	**3.81**	**2.88**	**7.73**	**3.92**	**4.85**	**S2 -> S3**	**δt_tx2(min)_**	**1.70**	**1.42**	**3.36**	**1.66**	**1.94**
	**δt_tx2(max)_**	**3.59**			**4.14**			**δt_tx2(max)_**	**1.92**			**1.44**	
**S4 -> S2**	**δt_tx2(min)_**	**3.81**		**6.75**	**2.94**	**3.87**	**S2 -> S4**	**δt_tx2(min)_**	**2.02**		**5.61**	**3.59**	**4.19**
	**δt_tx2(max)_**	**3.48**			**3.27**			**δt_tx2(max)_**	**2.46**			**3.15**	
**S4 -> S3**	**δt_tx2(min)_**	**3.38**		**4.39**	**1.01**	**1.51**	**S2 -> S5**	**δt_tx2(min)_**	**2.24**		**8.17**	**5.93**	**6.75**
	**δt_tx2(max)_**	**3.16**			**1.23**			**δt_tx2(max)_**	**2.68**			**5.49**	
**Earthquake Scenario 6**							**Earthquake Scenario 5**						
**S4 -> S1**	**δt_tx2(min)_**	**2.77**	**1.84**	**7.18**	**4.41**	**5.34**	**S2 -> S3**	**δt_tx2(min)_**	**1.60**	**1.32**	**3.36**	**1.76**	**2.04**
	**δt_tx2(max)_**	**2.55**			**4.63**			**δt_tx2(max)_**	**1.82**			**1.54**	
**S4 -> S2**	**δt_tx2(min)_**	**2.77**		**5.6**	**2.83**	**3.76**	**S2 -> S4**	**δt_tx2(min)_**	**1.92**		**5.41**	**3.49**	**4.09**
	**δt_tx2(max)_**	**2.44**			**3.16**			**δt_tx2(max)_**	**2.36**			**3.05**	
**S4 -> S3**	**δt_tx2(min)_**	**2.34**		**4.61**	**2.27**	**2.77**	**S2 -> S5**	**δt_tx2(min)_**	**2.14**		**8.01**	**5.87**	**6.69**
	**δt_tx2(max)_**	**2.12**			**2.49**			**δt_tx2(max)_**	**2.58**			**5.43**	

**Figure A1:**
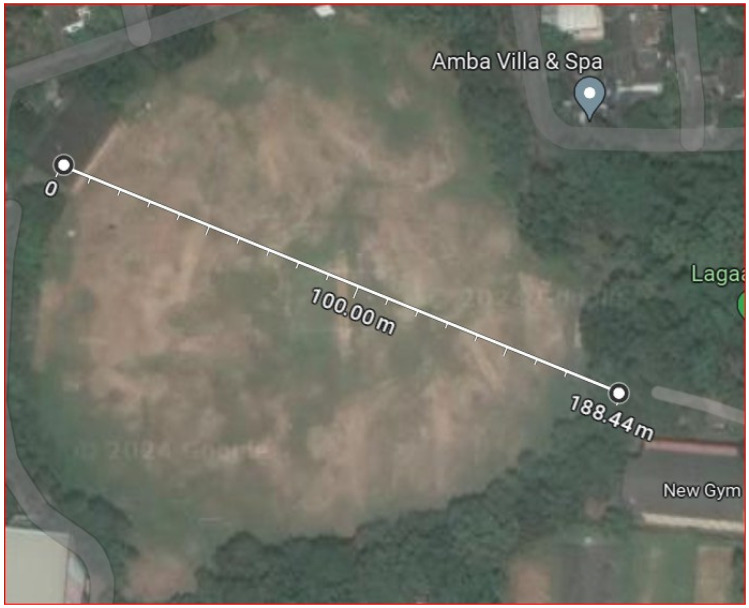
Map of area used for estimating $PL\left(d_{0}\right)$.

**Figure A2:**
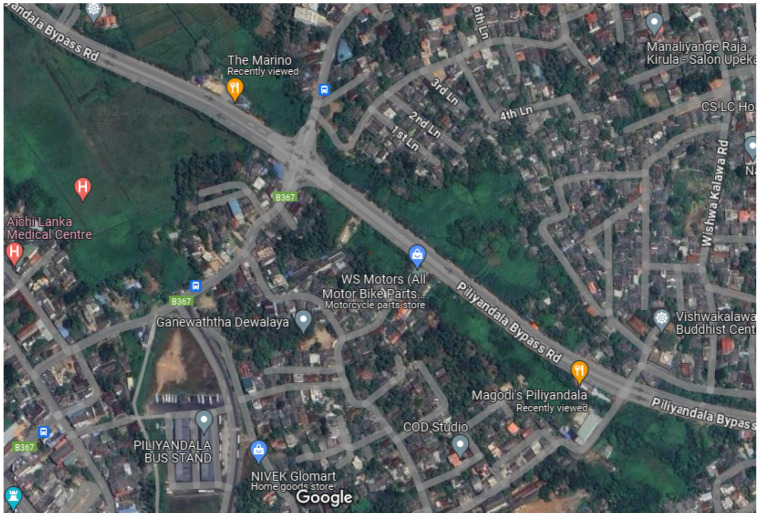
Map of area used for estimating propagation model parameters α and σ. Distances covered for measurements: 490 m for SF = 8, 764 m for SF = 12. Transmit power = 2 dBm.

## Data Availability

Developed by the authors [70] provides access to the GitHub repository containing the extended FLoRa framework. Available online: https://github.com/LoRaFYP19/omnetpp-mesh-tester.git.

## Appendix A. Estimation of Propagation Model Parameters

Estimation methodology:

*Step 1: *$PL\left(d_{0}\right)$ was estimated by averaging a series of RSSI measurements taken by transmitting 500 packets between a transmitter and a receiver separated by 190 m in the location shown in Figure A1 ($d_{0}=190$ m, approximately). The transmit power was set to 2 dBm.
*Step 2: *RSSI measurements were taken with an SF of 8 in the area shown in Figure A2. α and σ were computed based on this data following [59]. A distance up to 490 m was covered.
*Step 3: *The model parameters derived in Step 2 above were used to verify the model with a dataset taken with an SF of 12 in the same area. A distance up to 764 m were considered.

## Appendix B. Case Study EQ Scenarios

**Scenario**	**Epicentre Location**	
1	−38.89, 178.54	
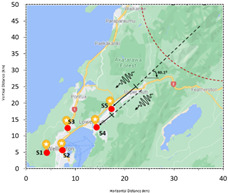
2	−30.15, −176.77	
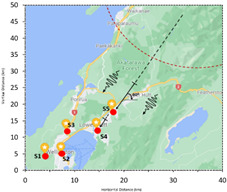
3	−43.583, 172.68	
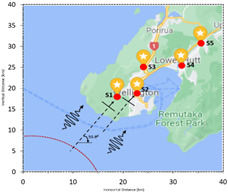
4	−41.61, 174.33	
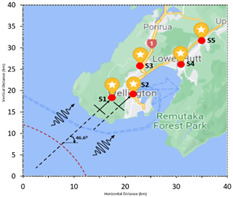
5	−42.757,173.077	
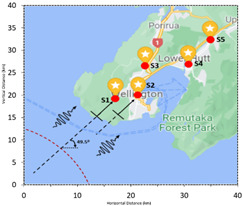
6	−41.20, 175.20	
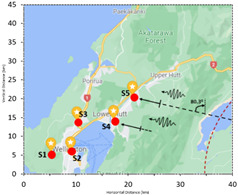

## Appendix C. Simulation Results for Transmission of Unverified Alerts with LoRa Multi-Hop Network (${\delta t}_{tx1}$)

**Transmission of Unverified Alert from S1 -> S2**				**Transmission of Unverified Alert from S5 -> S4**		
**No. of Hops**	**Total Distance (m)**	***δt_tx1_* (s)**		**No. of Hops**	**Total Distance (m)**	***δt_tx1_* (s)**

2	5733	0.218		4	9806	0.436
2	4985	0.218		4	10,255	0.436
2	5827	0.218		3	7849	0.327
2	5345	0.218		3	7803	0.327
2	5008	0.218		4	7794	0.436
2	4881	0.218		4	7790	0.436
3	4894	0.327		3	7793	0.327
3	4894	0.327		3	7793	0.327
2	4927	0.218		3	8009	0.327
2	4985	0.218		4	9145	0.436
2	4900	0.218		3	8787	0.327
2	4973	0.218		4	8437	0.436
2	5084	0.218		3	7898	0.327
2	6323	0.218		4	10,391	0.436
2	4989	0.218		4	10,591	0.436
3	5830	0.327		4	7794	0.436
3	4934	0.327		4	7956	0.436
2	4934	0.218		3	7971	0.327
2	5080	0.218		4	8170	0.436
2	4994	0.218		4	9612	0.436
2	5010	0.218		4	8958	0.436
3	6309	0.327		3	7815	0.327
2	5547	0.218		4	7817	0.436
3	5138	0.327		3	7819	0.327
2	3540	0.210		3	6589	0.327

## Appendix D. Simulation Results for EEW Transmission at S2 with LoRa Multi-Hop Network (${\delta t}_{tx2}$)

**LoRa Simulation Results for Earthquake Verification and EEW Generation at S2**								
**Transmission Delay S2 -> S3**			**Transmission Delay S2 -> S4**			**Transmission Delay S2 -> S5**		
**No. of Hops**	**Total Distance (m)**	***δt_tx2_* (s)**	**No. of Hops**	**Total Distance (m)**	***δt_tx2_* (s)**	**No. of Hops**	**Total Distance (m)**	***δt_tx2_* (s)**
4	7672	0.436	7	14,110	0.763	11	21,341	1.200
4	7675	0.436	10	14,831	1.091	10	20,967	1.091
4	7881	0.436	7	13,863	0.763	10	21,675	1.091
4	7859	0.436	9	14,255	0.982	10	21,277	1.091
3	8971	0.327	7	14,834	0.763	10	21,296	1.091
4	7972	0.436	7	15,363	0.763	10	21,136	1.091
3	9004	0.327	7	15,574	0.763	9	21,156	0.982
3	7701	0.327	7	15,593	0.763	9	21,011	0.982
3	7681	0.327	7	13,386	0.763	9	21,147	0.982
4	7720	0.436	7	16,131	0.763	9	21,236	0.982
3	8506	0.327	8	15,348	0.872	10	21,185	1.091
4	9114	0.436	9	14,597	0.982	11	22,425	1.200
4	8042	0.436	7	14,145	0.763	10	20,948	1.091
4	7735	0.436	8	13,954	0.872	10	21,164	1.091
5	8163	0.545	9	17,608	0.982	11	21,583	1.200
3	7694	0.327	7	14,783	0.763	10	21,710	1.091
5	9358	0.545	9	16,041	0.982	12	21,391	1.309
3	7685	0.327	6	14,075	0.654	9	21,113	0.982
5	7872	0.545	8	14,308	0.872	11	21,358	1.200
4	8375	0.436	8	17,021	0.872	10	21,518	1.091
5	7727	0.545	8	16,050	0.872	10	21,239	1.091
4	7778	0.436	7	14,746	0.763	9	22,272	0.982
3	7903	0.327	7	15,166	0.763	10	22,028	1.091
5	8909	0.545	9	14,488	0.982	12	20,927	1.309
3	6814	0.327	6	10,016	0.654	8	16,415	0.872

## Appendix E. Simulation Results for EEW Transmission at S2 with LoRa Multi-Hop Network (${\delta t}_{tx2})$

**LoRa Simulation Results for Earthquake Verification and EEW Generation at S4**								
**Transmission Delay S4 -> S2**			**Transmission Delay S4 -> S2**			**Transmission Delay S4 -> S3**		
**No. of Hops**	**Total Distance (m)**	***δt_tx2_* (s)**	**No. of Hops**	**Total Distance (m)**	***δt_tx2_* (s)**	**No. of Hops**	Total Distance (m)	***δt_tx2_* (s)**
9	18,745	0.982	7	14,079	0.763	4	9272	0.436
7	17,592	0.763	7	16,618	0.763	3	9187	0.327
9	17,595	0.982	8	13,524	0.873	4	10,994	0.436
8	17,650	0.873	7	13,733	0.763	4	9196	0.436
8	19,009	0.873	9	15,802	0.982	4	10,158	0.436
9	17,592	0.982	7	16,066	0.763	4	10,821	0.436
9	17,592	0.982	7	15,645	0.763	4	10,655	0.436
9	17,592	0.982	7	15,645	0.763	4	10,655	0.436
8	17,758	0.873	7	15,001	0.763	5	9476	0.545
7	18,453	0.763	7	13,531	0.763	4	9343	0.436
9	17,955	0.982	8	16,288	0.873	5	9228	0.545
9	17,617	0.982	9	19,101	0.982	5	9269	0.545
8	18,997	0.873	7	17,071	0.763	4	9281	0.436
8	17,761	0.873	8	17,837	0.873	5	10,512	0.545
9	19,936	0.982	9	17,662	0.982	4	10,856	0.436
8	19,009	0.873	9	15,802	0.982	4	10,158	0.436
8	17,739	0.873	9	14,881	0.982	4	9348	0.436
7	18,038	0.763	6	14,896	0.654	4	9272	0.436
7	18,088	0.763	8	17,483	0.873	4	9335	0.436
8	19,454	0.873	8	15,078	0.873	5	9905	0.545
7	18,363	0.763	8	18,496	0.873	4	9425	0.436
8	17,824	0.873	7	15,767	0.763	4	9324	0.436
8	18,559	0.873	7	15,072	0.763	4	9314	0.436
8	17,739	0.873	9	16,294	0.982	4	9195	0.436
7	13,266	0.763	6	10,016	0.654	3	6203	0.327
